# A viral-fusion-peptide-like molecular switch drives membrane insertion of botulinum neurotoxin A1

**DOI:** 10.1038/s41467-018-07789-4

**Published:** 2018-12-18

**Authors:** Kwok-ho Lam, Zhuojun Guo, Nadja Krez, Tsutomu Matsui, Kay Perry, Jasmin Weisemann, Andreas Rummel, Mark E. Bowen, Rongsheng Jin

**Affiliations:** 1 Department of Physiology and Biophysics, University of California, Irvine, 92697 CA USA; 2 Department of Physiology and Biophysics, Stony Brook University, Stony Brook, 11794 NY USA; 3 Institut für Toxikologie, Medizinische Hochschule Hannover, Hannover, 30623 Germany; 4 Stanford Synchrotron Radiation Lightsource, SLAC National Accelerator Laboratory, Stanford University, Menlo Park, 94025 CA USA; 5 NE-CAT and Department of Chemistry and Chemical Biology, Argonne National Laboratory, Cornell University, Argonne, 60439 IL USA

## Abstract

Botulinum neurotoxin (BoNT) delivers its protease domain across the vesicle membrane to enter the neuronal cytosol upon vesicle acidification. This process is mediated by its translocation domain (H_N_), but the molecular mechanism underlying membrane insertion of H_N_ remains poorly understood. Here, we report two crystal structures of BoNT/A1 H_N_ that reveal a novel molecular switch (termed BoNT-switch) in H_N_, where buried α-helices transform into surface-exposed hydrophobic β-hairpins triggered by acidic pH. Locking the BoNT-switch by disulfide trapping inhibited the association of H_N_ with anionic liposomes, blocked channel formation by H_N_, and reduced the neurotoxicity of BoNT/A1 by up to ~180-fold. Single particle counting studies showed that an acidic environment tends to promote BoNT/A1 self-association on liposomes, which is partly regulated by the BoNT-switch. These findings suggest that the BoNT-switch flips out upon exposure to the acidic endosomal pH, which enables membrane insertion of H_N_ that subsequently leads to LC delivery.

## Abstract (web summary)

The translocation domain (H_N_) of Botulinum neurotoxins (BoNTs) mediates the delivery of the BoNT light chain (LC) into neuronal cytosol. Here the authors provide insights into H_N_ membrane insertion by determining the crystal structure of BoNT/A1 H_N_ at acidic pH, which reveals a molecular switch in H_N_, where buried α-helices are transformed into surface-exposed hydrophobic β-hairpins.

## Introduction

Many bacterial toxins and viruses gain access to intracellular targets in their hosts by exploiting the endocytic pathway, such as anthrax toxin^1^, diphtheria toxin^2^, Ebola virus^3^, and vesicular stomatitis virus^4^. Some of the toxins and viruses detect acidification of the vesicle lumen and subsequently change from a water-soluble form to a membrane-embedded form to exert their functions. Botulinum neurotoxins (BoNTs), the most poisonous biological toxins for mammals, are speculated to exploit a similar mechanism to block neurotransmitter release at neuromuscular junctions that results in fatal botulism^5^. As some BoNTs, in particular type A1 (BoNT/A1), are powerful medicines as well as potential bio-weapons^6,7^, a better understanding of the molecular mechanism underlying BoNT/A1 membrane translocation has great potential for improving its therapeutic efficacy and developing antitoxins.

**Fig. 1:**
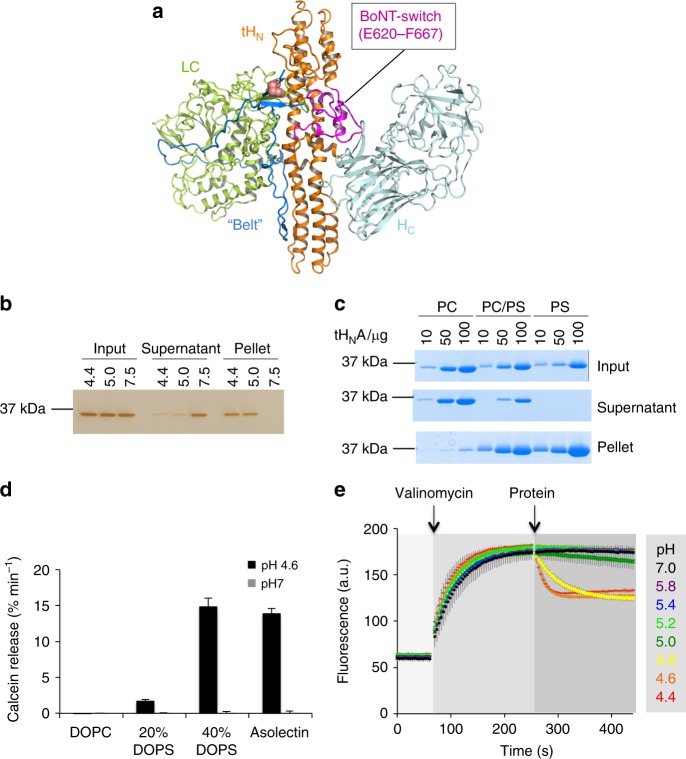
Biochemical characterization of tH_N_A. **a** The structure of BoNT/A1 (PDB code 3BTA). LC, the “belt”, tH_N_, and H_C_ are colored green, marine, orange, and cyan, respectively. The disulfide linkage between LC and H_N_ is shown as salmon sphere and the BoNT-switch is highlighted in magenta. **b**, **c** Co-sedimentation of tH_N_A with liposomes. tH_N_A was incubated with asolectin liposomes at pH 7.5, 5.0 or 4.4 (**b**); or incubated with liposomes containing 60/40 mol% PC/cholesterol, 30/30/40 mol% PC/PS/cholesterol, or 60/40 mol% PS/cholesterol at pH 4.4 (**c**). After liposomes were pelleted, the proteins in input, supernatant, and pellet fractions were analyzed by SDS-PAGE. These experiments were performed in triplicate and quantification of protein band intensities are shown in Supplementary Fig. 11. Uncropped images of gels are shown in Supplementary Fig. 13. **d** Calcein dye release assay. tH_N_A was tested with four different liposomes loaded with 50 mM calcein at pH 4.6 or pH 7.0, whereas liposomes were composed of DOPC alone, 80/20 mol% DOPC/DOPS, 60/40 mol% DOPC/DOPS, or asolectin. The rate of calcein dye release was determined based on the increase of fluorescence at 525 nm during excitation at 493 nm. Error bars indicate SD of triplicate measurements. **e** Membrane depolarization assay. Liposomes composed of 70/20/10 mol% DOPC/DOPS/cholesterol were polarized at a positive internal voltage by adding valinomycin in the presence of a transmembrane KCl gradient. Membrane potential was measured using the voltage-sensitive fluorescence dye ANS. After 3 min, tH_N_A was added at the indicated buffer pH. The data are presented as mean ± S.D., *n* = 3

There are seven established BoNT serotypes, designated as BoNT/A to G, which include more than 40 subtypes^8^. BoNTs are members of single-chain AB toxins that also include diphtheria toxin and tetanus neurotoxin^9^. A BoNT molecule consists of a light chain (LC) and a heavy chain (HC) that are linked by a disulfide bridge^10–12^. LC is a zinc-dependent endopeptidase that is delivered into the cytosol where it degrades SNARE proteins to inhibit synaptic vesicle exocytosis. Membrane translocation of LC is mediated by HC, which can be subdivided into a translocation domain (H_N_) and a receptor-binding domain (H_C_) (Fig. 1a). The N-terminus of H_N_ forms an extended “belt” that wraps around the LC and links H_N_ to LC via a disulfide bond^13^. The BoNT H_C_ binds a specific protein receptor and a ganglioside on the neuronal surface to enable toxin endocytosis^14–19^.

The mechanisms underlying BoNT membrane insertion and LC translocation have been the subject of considerable debates. Earlier studies showed that BoNT/A1 translocated LC in planar lipid bilayer in the presence of a transmembrane pH gradient, an electrochemical potential, and a redox gradient^20,21^. It was proposed that acidic pH might induce a structural change of BoNT and promote its interaction with phospholipids^17^, which is similar to other pore forming toxins^2,22,23^. Although no conformational change was detected in BoNT at acidic pH in the available crystal structures^24^, it underwent an unknown structural rearrangement after binding to ganglioside-containing membranes^25–29^. Furthermore, the H_N_ core lacking the “belt” (termed tH_N_ thereafter, Fig. 1a) was shown to form an ion channel independent of pH, and no clear structural change was detected by spectroscopic methods at acidic pH^30–32^. Other regions of H_N_ have also been proposed to be involved in membrane insertion^33,34^. However, the structural rearrangement of BoNT during LC delivery, particularly the trigger for membrane insertion of H_N_, has not been visualized.

To better understand the mechanism of BoNT membrane translocation, we determined the crystal structure of tH_N_ of BoNT/A1 (tH_N_A, residues K547–K871) at pH 5.1, as well as a point mutation of tH_N_A (tH_N_A^F658E^) at pH 8.5. To our surprise, these two structures revealed a previously unrecognized amphipathic region of H_N_ (residues E620–F667, termed the BoNT-switch) that undergoes a profound pH-dependent structural rearrangement to form surface-exposed β-hairpins. Furthermore, the BoNT-switch shares a high sequence similarity with the internal fusion loop (IFL) of Ebola virus glycoprotein 2. A combination of structural studies, biochemical assays, single-molecule subunit counting, and functional assays confirmed that, upon endosome acidification, the BoNT-switch triggers the transformation of BoNT/A1 into a transmembrane state for LC delivery, which is essential for its extreme toxicity.

## Results

### Acidic pH triggers membrane insertion of tH_N_A

The H_N_ of BoNTs has evaded structural studies due to problematic protein folding during recombinant expression^31,32^. We and others found that H_N_A primarily formed inclusion bodies in *Escherichia coli*, which could be refolded when stabilized by detergent. However, we found that the beltless H_N_ (termed tH_N_A) could be successfully expressed in soluble form and purified to high homogeneity (Supplementary Fig. 1a). Size-exclusion chromatography-coupled small-angle X-ray scattering (SEC-SAXS) showed that tH_N_A was monomeric at neutral pH and displayed a conformation comparable to that observed in the crystal structure of full-length BoNT/A1^10^ (Supplementary Fig. 1b, c). Thus, deletion of the belt region improves protein folding of H_N_ without altering its tertiary structure.

Some earlier studies suggested that low pH and anionic phospholipids may promote the membrane interaction of H_N_^30–32,35^. We conducted a liposome co-sedimentation assay to investigate the effect of pH and lipid composition on tH_N_A–membrane association. We found that tH_N_A was co-pelleted with asolectin liposomes at pH 4.4 or 5.0, but not at pH 7.5, indicating that acidic pH enhances the association of tH_N_A with lipid (Fig. 1b). We then examined liposomes containing different ratios of anionic phosphatidylserine (PS) and neutral phosphatidylcholine (PC) lipids and found that tH_N_A binding to liposomes at pH 4.4 was greatly enhanced by increasing concentrations of PS. This demonstrates that tH_N_A shows selectivity for anionic lipids (Fig. 1c).

We further characterized the membrane insertion of tH_N_A using two complementary assays. First, we monitored the ability of tH_N_A to permeabilize calcein-entrapped liposomes. We found that tH_N_A increased the rate of calcein release at acidic pH when liposomes contained the anionic phospholipid 1,2-dioleoyl-sn-glycero-3-phospho-L-serine (DOPS) or asolectin, but not when liposomes contained only the neutral lipid 1,2-dioleoyl-sn-glycero-3-phosphocholine (DOPC) (Fig. 1d). We then studied the ability of tH_N_A to dissipate valinomycin-induced membrane potential in liposomes containing 20% DOPS at different pH (Fig. 1e)^36,37^, and found that tH_N_A induced membrane depolarization at acidic pH ( < 5.0). It is worth noting that this pH value is close to the estimated luminal surface pH of synaptic vesicles, which could be one unit lower than that inside the lumen (~5.5)^38,39^. These results consistently demonstrate that tH_N_A favorably interacts and inserts into membranes containing anionic lipids at acidic pH.

### Acidic pH triggers a structural rearrangement of tH_N_A

**Fig. 2:**
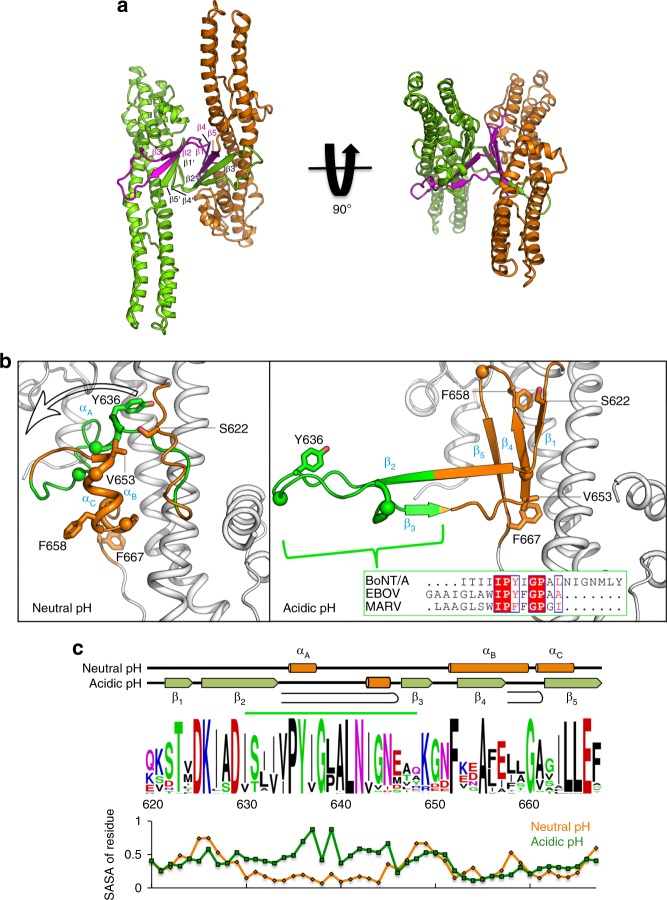
Crystal structure of tH_N_A at an acidic pH. **a** Structure of a tH_N_A dimer (orange and green) observed in an asymmetric unit. The BoNT-switch of chain A is colored in magenta. **b** The structures of the BoNT-switch at neutral (left) and acidic pH (right). The β2/β3 loop is highlighted in green. Residues discussed in the text are shown as sticks, Cα atoms of conserved glycines are drawn in spheres. (Boxed) Sequence alignment of the β2/β3 loop and the internal fusion loops of Ebola virus (EBOV) and Marburg virus (MARV) glycoproteins were generated with ESPript 3.0. **c** Amino acid sequences of E620-F667 (BoNT/A1 numbering) from 42 BoNT subtypes^8^, TeNT, BoNT/X, and eBoNT/J were aligned (Clustal Omega) and depicted as sequence logo. Individual sequences are shown in Supplementary Fig. 6. The secondary structure of the BoNT/A1-switch at neutral and acidic pH is shown on the top. The β2/β3 loop is underlined in green. The solvent-accessible surface area (SASA) of the corresponding residues is shown at the bottom. SASA was calculated using the program POPS^42^. The SASA values are for the entire residue and represent fraction of exposed surface area

To better understand how acidic pH triggers membrane insertion of tH_N_A, we crystallized tH_N_A at pH 5.1 and determined the structure at 2.7 Å resolution (Methods) (Table 1). Two molecules of tH_N_A form a homodimer in the asymmetric unit (Fig. 2a). Each tH_N_A is composed of two long coiled-coil helices that are wrapped around by several shorter helices at the two ends, and the overall structure of tH_N_A is similar to that observed in full-length BoNT/A1 crystallized at a neutral pH^10^. However, we observed a major conformational change in a central helical bundle (residues E620–F667) of H_N_. This region, which we term the “BoNT-switch”, is composed of disordered loops and three short helices (α_A_–α_C_) at neutral pH, but changes into five β strands (β1–β5) at acidic pH (Fig. 2b).

**Table 1:** Data collection and refinement statistics

	tH_N_A	tH_N_A^F658E^
Data collection		
Space group	H 3 2	P 63 2 2
Cell dimensions		
* a*, *b*, *c* (Å)	167.66, 167.66, 222.83	158.27, 158.27, 123.97
* α*, *β*, *γ* (°)	90, 90, 120	90, 90, 120
Resolution (Å)	52.01–2.70 (2.83–2.70)^a^	42.87–3.02 (3.20–3.02)
R_merge_	0.064 (0.609)	0.093 (0.616)
CC1/2	0.999 (0.711)	0.999 (0.886)
I/σ(I)	12.8 (2.0)	15 (3.6)
Completeness (%)	99.3 (99.1)	99.9 (100)
Redundancy	4.6 (4.1)	9.1 (9.3)
Refinement		
Resolution (Å)	52.01–2.70	42.87–3.02
No. reflections	32906	18514
Reflections used for R_free_	1662	946
R_work_/R_free_	0.209 / 0.223	0.182 / 0.237
No. atoms	5176	2647
Protein	5144	2602
Ligand/ion	5	45
Water	27	—
B-factors	85.70	83.40
Protein	85.80	83.00
Ligand/ion	117.60	107.10
Water	65.50	—
R.m.s. deviations		
Bond lengths (Å)	0.009	0.010
Bond angles (°)	1.167	1.116

One crystal was used for each structure

^a^Statistics for the highest resolution shell are shown in parentheses

At neutral pH, α_A_ helix and the surrounding loops (residues I630–Y648, named β2/β3 loop thereafter, labeled green in Fig. 2b) are partially buried in a hydrophobic groove located between α_B_ (residues F652–S659) and the coiled-coil helices (Fig. 2b). The conformation of this region is similar among BoNT/B, D, E, and TeNT. These contacts are stabilized by Y636 that interacts with F585, M732, and I784 (Fig. 2b). At acidic pH, the α_A_ protrudes outward to form the center of the β2/β3 hairpin. This leaves the hydrophobic groove exposed to solvent, thus helices α_B_ and α_C_ transform into the β4/β5 hairpin to partly shield the solvent-exposed surface. The low pH conformation is facilitated by several non-polar residues (V653, F658, and F667). These residues are solvent-exposed at neutral pH, but flip inward at acidic pH. Notably, the highly conserved Y636 that is important for stabilizing the local structure at neutral pH flips out to be solvent exposed at acidic pH. Interestingly, the original position of Y636 is taken over by F658. This finding suggests that F658 may contribute to the pH-dependent conformational transition. Additionally, the BoNT-switch contains several glycine residues (G638, G644, G654, and G660) that likely provide the conformational flexibility to accommodate such a dramatic structural transition (Fig. 2b). Interestingly, the Propka algorithm^40^ predicted that three acidic residues (E620, D629, and E666) located within the BoNT-switch have increased pKa values in the context of the neutral pH conformation^38^. Two of these residues, D629 and E666, are locked in hydrogen bonds to S794 and Y250, respectively, while these interactions are released at acidic pH (Supplementary Fig. 2). Therefore, we suspect that these titratable charged residues could be involved in sensing low pH.

As this is the only crystal structure for the isolated tH_N_A and all the other available structures are for the full-length BoNT/A or LC/A-H_N_A^10,41^, it would be informative for structural analysis if we could obtain the structure of tH_N_A, without the influence of other BoNT/A domains, at a neutral pH. Interestingly, diffraction-quality crystals of tH_N_A could only be obtained at an acidic pH despite thorough crystallization screens at various pH. We suspected that the isolated tH_N_A domain may be prone to adopting the acidic pH conformation needed for LC translocation, and its neutral pH conformation could be stabilized by LC/A and/or H_C_A. Taking advantage of our new crystal structure, we set out to look for point mutations on tH_N_A that shift the structural equilibrium of tH_N_A more towards the neutral pH conformation as opposed to the acidic pH conformation. After extensive search and screening, we found that tH_N_A carrying a single point mutation of F658E could be crystallized at pH 8.5 and we determined its structure at 3.02 Å (Table 1 and Supplementary Fig. 3a). This result is consistent with our structural analysis that suggested F658E may destabilize the acidic pH conformation, but does not directly affect the neutral pH conformation (Fig. 2b). We found that the structure of tH_N_A^F658E^ is highly similar to the corresponding region in full-length BoNT/A that was crystallized at a neutral pH (root-mean-square deviation of ~0.96 Å). Notably, the BoNT-switch adopts an almost identical structure in tH_N_A^F658E^ and the full-length toxin (Supplementary Fig. 3b). Taken together, the structures of tH_N_A at pH 5.1 and tH_N_A^F658E^ at pH 8.5 allow us to directly visualize how the environmental pH regulates the conformation of the BoNT-switch.

### The BoNT-switch is similar to the Ebola GP2 fusion loop

The structural rearrangement of the BoNT-switch causes a ~21% increase in the solvent-accessible hydrophobic surface in tH_N_A^42^, which is largely ascribed to the formation of the β2/β3 loop (Fig. 2b, c). The structural and sequence analyses of the BoNT-switch indicate that the solvent-exposed β2/β3 loop is favorable for interacting with lipids, as supported by the hydropathy analysis of the BoNT/A sequence^43,44^ (Supplementary Fig. 4). Interestingly, these hydrophobic patches in β2/β3, which are energetically not favored in an aqueous environment, are shielded by forming a homodimer in the crystal lattice, which buries a surface area of ~2,625 Å^2^ per molecule (PDBePISA). Specifically, the tH_N_A homodimer forms a two-side flattened β-barrel that is stabilized by swapping the β2/β3 hairpin between the two protomers, which forms an antiparallel β sheet with β1, β4, and β5 (Fig. 2a). Dimer formation is stabilized by hydrogen bonds primarily involving the main-chain atoms of β2 and β5 (Supplementary Fig. 5a). As a result, most of the exposed hydrophobic side-chains of the β-hairpins are buried within the tH_N_A homodimer in the crystal structure (Supplementary Fig. 5b, c).

Remarkably, we found that the β2/β3 loop contains a stretch of hydrophobic residues rich in aliphatic side-chains, which shares a high sequence similarity to a lipid-binding peptide of the glycoprotein 2 (GP2) IFL of Ebola virus, a member of the Filoviridae family of enveloped viruses. IFL is known to control viral-host membrane fusion at low endosomal pH by directly engaging the host membrane^45^ (Fig. 2b). The loops in both BoNT/A1 and GP2 contain an “aromatic-hydrophobic-glycine” tripeptide motif flanked by two proline residues, which would favorably interact with the interfacial region of lipid bilayers^46^. Amino acid sequence alignments among the 44 known BoNT subtypes, including the recently identified BoNT/HA^47^, BoNT/X^48^, and eBoNT/J (aka BoNT/En)^49,50^, as well as TeNT, showed that the sequence of the β2/β3 loop is highly conserved, suggesting a functional role for this loop in BoNTs (Fig. 2c). We noted that the second proline (P639) is conserved among BoNT/A, C, D, G, X, and TeNT, but this residue is replaced by a leucine in BoNT/B, E, F, and HA or an asparagine in eBoNT/J (Supplementary Fig. 6). Such variation may indicate that the structure of the BoNT-switch when inserted into membrane could be different from that of the viral-fusion-loop of GP2.

### The BoNT-switch is important to the membrane insertion of H_N_

**Fig. 3:**
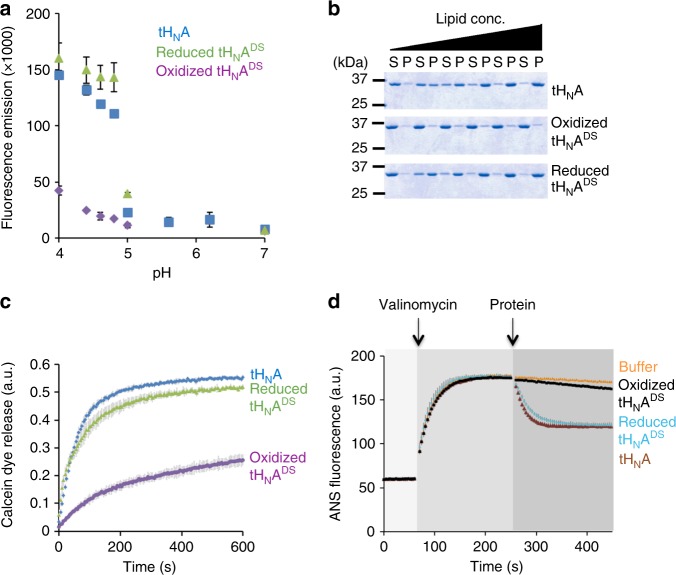
Modulating the conformation of the BoNT-switch using an engineered disulfide bond trapping. tH_N_A^DS^ was analyzed by four independent assays: **a** ANS binding; **b** liposome association: liposome co-sedimentation was conducted by incubating the WT tH_N_A or tH_N_A^DS^ with different concentrations of liposomes containing 20% DOPS and 80% OBPC. Supernatant (S) and pellet (P) fractions were analyzed by SDS-PAGE. Quantification of protein band intensities are shown in Supplementary Fig. 12. Uncropped images of gels are shown in Supplementary Fig. 13; **c** calcein dye release; and **d** membrane depolarization assay. 5 mM TCEP was added for the reaction of reduced tH_N_A^DS^ and no reducing agent was used for wild type or oxidized tH_N_A^DS^. The data are presented as mean ± S.D., *n* = 3

To further characterize the role of the BoNT-switch in membrane insertion of tH_N_A, we engineered a covalent tethering between loop_A_ and α_B_ by replacing S622 and V653 with cysteines (named tH_N_A^DS^) (Fig. 2b and Supplementary Fig. 7). The crystal structures showed that these two solvent-exposed residues are 5.3 Å apart (Cβ–Cβ distance) when BoNT/A1 is at a neutral pH, but they move away from each other (15.5 Å) upon the conformational change induced by acidic pH. Therefore, the disulfide bridge between S622C and V653C will inhibit the helix-to-strand transition of α_B_ when oxidized, and the inhibition will be released when reduced. To monitor exposure of hydrophobic surface in tH_N_A, we used 8-anilinonaphthalene-1-sulfonic acid (ANS) as an indicator that displays increased fluorescence intensity when it binds to hydrophobic patches in a protein (Fig. 3a). We found that ANS intensity in the presence of tH_N_A remained unchanged at pH 5–7, but dramatically increased at pH below 5.0. This is consistent with our structural data showing exposure of hydrophobic surface in tH_N_A at acidic pH. The transitional pH for tH_N_A (~4.82) is similar to that reported previously for full-length BoNT/A (~4.55)^29^. We then asked whether the structural change in tH_N_A^DS^ could be regulated by the engineered disulfide bond. The reduced tH_N_A^DS^ showed an acidic-pH-dependent increase in ANS fluorescence that was similar to the WT tH_N_A, indicating that the cysteine mutations on S622 and V653 did not affect the BoNT-switch. In contrast, the oxidized tH_N_A^DS^ did not exhibit a pH-dependent change in ANS fluorescence as there was only a subtle intensity increase at acidic pH. Therefore, the engineered disulfide linkage prevented the acid-induced release of the BoNT-switch.

In a parallel approach, we performed a fluorescence-based thermal shift assay to examine how the disulfide bridge affects the structure and stability of tH_N_A^DS^ (Supplementary Fig. 8). At neutral pH, the melting temperature (Tm) of the oxidized tH_N_A^DS^, but not the reduced tH_N_A^DS^, increased by ~15% relative to WT tH_N_A, indicating that the engineered disulfide bond stabilized tH_N_A. The Tm could not be measured at pH 4.6 for either WT tH_N_A or the reduced tH_N_A^DS^ due to high fluorescence intensity, because large hydrophobic surfaces in tH_N_A were exposed at acidic pH. However, the Tm of the oxidized tH_N_A^DS^ could be measured at pH 4.6, which only decreased by ~6% in comparison to the Tm at neutral pH, suggesting that the oxidized tH_N_A^DS^ is trapped in the neutral-pH-like conformation and insensitive to pH changes. These findings demonstrate that the BoNT-switch regulates the exposure of hydrophobic surface at acidic pH.

Using tH_N_A^DS^ as a unique molecular probe, we further examined lipid association of tH_N_A using a liposome co-sedimentation assay (Fig. 3b). The WT tH_N_A and the reduced tH_N_A^DS^ co-sedimented with anionic liposomes containing 20% DOPS at pH 4.6 in a lipid concentration-dependent fashion. In contrast, association of the oxidized tH_N_A^DS^ with liposomes was significantly reduced at all lipid concentrations tested. Furthermore, the oxidized tH_N_A^DS^ exhibited a marked decrease in both the rate of calcein dye release and the ability to dissipate the membrane potential of a polarized membrane in liposomes, while the reduced tH_N_A^DS^ behaved like the WT tH_N_A (Fig. 3c, d). These findings prove that the acidic pH induced structural change of the BoNT-switch is necessary for tH_N_A to transform from a water-soluble state to a transmembrane state.

### The BoNT-switch regulates self-association of BoNT/A1

Since the tH_N_A homodimer observed in the crystal lattice could be affected by crystal packing, we used analytical size-exclusion chromatography to examine the effect of pH on self-association of tH_N_A in solution. We found that tH_N_A shifted to a higher oligomeric state at acidic pH ( < 5.4) in a concentration-dependent manner (Supplementary Fig. 9a, b). Importantly, the oxidized tH_N_A^DS^ remained monomeric at pH 4.6, while the reduced form shifted to oligomers like the WT tH_N_A (Supplementary Fig. 9c). Therefore, tH_N_A self-association seems to be needed to protect the unique conformation of the BoNT-switch at acidic pH, whereas such protection could be provided by other BoNT domains in the context of the full-length toxin or by the vesicle membrane in vivo. Furthermore, the micromolar tH_N_A concentration used in this assay is much higher than the physiological concentration of BoNT/A1 at the neuromuscular junctions. Therefore, tH_N_A dimerization may not be directly relevant for BoNT/A1 function in vivo. In support of this, we found that the full-length BoNT/A1i (a genetically inactivated BoNT/A1 carrying R363A/Y366F mutations) does not exhibit pH-dependent self-association in solution (Supplementary Fig. 9d).

To investigate whether membrane binding may trigger BoNT/A1 self-association, we used single-molecule photobleaching to count the number of BoNT/A1 molecules recruited to single liposomes containing gangliosides. Photobleach step counting has been widely used to study protein oligomerization on the membrane^51–53^. Since BoNT/A1 contains several functionally important cysteine residues, we could not chemically-label BoNT/A1. To circumvent this, we fluorescently labeled a BoNT/A1-specific camelid heavy-chain-only antibody (ciA-D12) that binds to LC with high affinity (K_d_ ~0.2 nM) and does not affect BoNT/A1 function^54^. Importantly, the ciA-D12-BoNT/A1i interaction is not affected by acidic pH (Supplementary Fig. 10). An engineered cysteine residue (S142C) in ciA-D12 was labeled to > 99% efficiency with Alexa Fluor 647 maleimide (termed D12*). The D12*-BoNT/A1i complex was assembled and purified by size-exclusion chromatography.

**Fig. 4:**
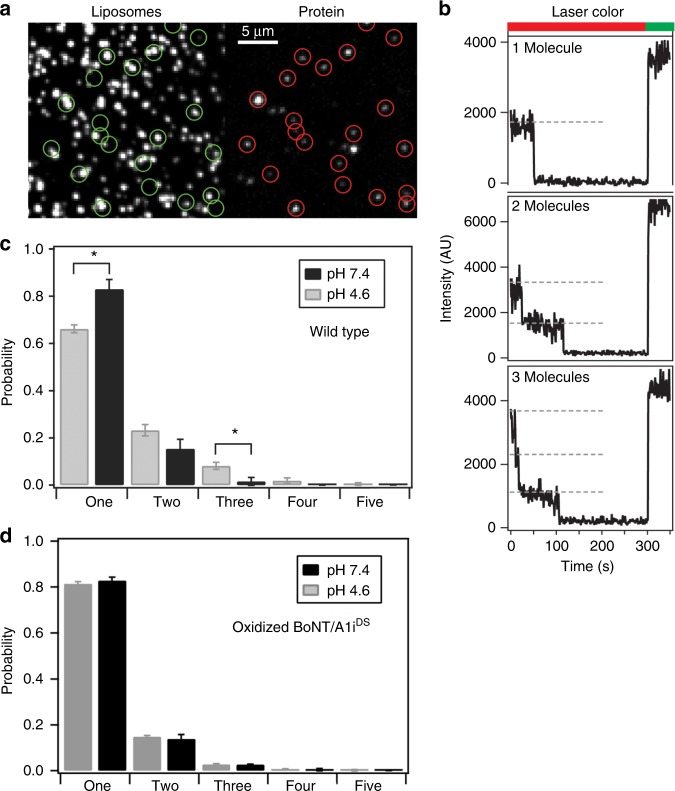
Single-molecule analysis of the distribution of BoNT/A1i on individual liposomes. **a** Representative TIRF microscope images showing co-localization of Alexa 647-labeled BoNT/A1i-D12* complexes (right) with rhodamine-labeled liposomes (left). In these experiments, < 10% of liposomes were occupied by protein to minimize co-localization. The circled spots on the right were used for photobleaching analysis only when co-localized with rhodamine fluorescence. **b** Representative time traces of fluorescence emission from spots used to assess the oligomerization state. The bar above the panel indicates the excitation with red laser used to first bleach the Alexa 647 followed by green laser to probe for rhodamine emission. Examples are shown for 1-, 2-, and 3-step photobleaching events. Dashed lines highlight individual bleaching steps. (**c**) Distribution of bleaching steps for BoNT/A1i-D12* at pH 4.6 (*n* = 935) and pH 7.4 (*n* = 482). The increase in 1-step events and decrease in 3-step events upon raising the pH to 7.4 were statistically significant (asterisk) with *p* = 0.0001 and 0.0065, respectively. The decrease in 2-step events was just above significance with *p* = 0.077. **d** Distribution of photobleaching steps for oxidized BoNT/A1i^DS^-D12* at pH 4.6 (*n* = 589) and pH 7.4 (*n* = 535). No significant differences were observed when the BoNT-switch was locked by a disulfide bond. Error bars indicate standard error of measurement (SEM) from replicate analysis

The BoNT/A1i-D12* complex was reconstituted on 100 nm anionic liposomes containing 10% polysialoganglioside GT1b. The liposomes also contained 0.5 mol% biotin-labeled lipids to allow liposomes to be attached to a streptavidin-coated microscope slide while unbound proteins were washed away by rinsing. To allow co-localization of protein and liposomes, we included 0.5 mol% Rhodamine-labeled lipids and imaged the samples with two-color Total Internal Reflection Fluorescence (TIRF) microscopy. Liposomes were deposited under dilute conditions to achieve optical resolution of individual liposomes, which appeared as diffraction-limited spots (Fig. 4a). Under our incubation conditions (10 nM protein with 0.5 mg/mL lipid), we estimated that ~10% of liposomes were occupied by protein molecules. The low surface density and low liposome occupancy both help to minimize the probability of false co-localization of proteins.

The number of BoNT/A1i-D12* complexes on a liposome was determined by counting Alexa Fluor 647 photobleaching steps for diffraction-limited spots that also contained Rhodamine emission (Fig. 4b). At pH 4.6, we found that a major fraction of BoNT/A1i-D12* fluorescent spots displayed single-step bleaching with a smaller fraction showing multi-step bleaching up to five steps (Fig. 4c). We observed 66.2 ± 1.7%, 23.2 ± 2.4%, 8.2 ± 1.5%, 1.9 ± 1.2%, and 0.5 ± 0.5% for one to five-step events, respectively. At neutral pH, we found a decrease in protein co-localization events. Specifically, two- and three-step events were reduced and higher order events disappeared entirely while the one-step bleaching events increased by 16.8 ± 3.8%. This finding suggests that, while BoNT/A1i binds to membranes predominantly as monomer at neutral or acidic pH on liposomes we tested here, an acidic environment increased the degree of BoNT/A self-association. Interestingly, low-pH-dependent self-association of BoNT was previously observed for BoNT/B on artificial membranes^27^.

To test whether locking the BoNT-switch could affect self-association of BoNT/A1 on membranes, we incorporated S622C and V653C into the full-length BoNT/A1i (termed BoNT/Ai^DS^). BoNT/Ai^DS^ was oxidized and then assembled with the labeled antibody D12*. We found that the oxidized BoNT/A1i^DS^ showed a similar liposome occupancy as the WT toxin, suggesting that locking the BoNT-switch did not significantly affect membrane association of BoNT/A1. We then examined the oligomerization state of BoNT/Ai^DS^ by analyzing the photobleaching time traces as described above. Notably, locking the BoNT-switch showed significant effect on oligomerization of the full-length toxin (Fig. 4d). The increase in protein oligomerization at pH 4.6 was completely eliminated in the oxidized BoNT/A1i^DS^, even though BoNT/A1i^DS^ showed the same level of monomers at pH 7.4 as the WT toxin (82.8 ± 1.5%). Taken together, we found that acidic pH tends to promote BoNT/A1 oligomerization on liposomes, which is partly regulated by the BoNT-switch. The physiological relevance of BoNT/A1 self-association on the vesicle membrane in vivo warrants further investigation.

### The BoNT-switch is crucial to the extreme potency of BoNT/A1

**Fig. 5:**
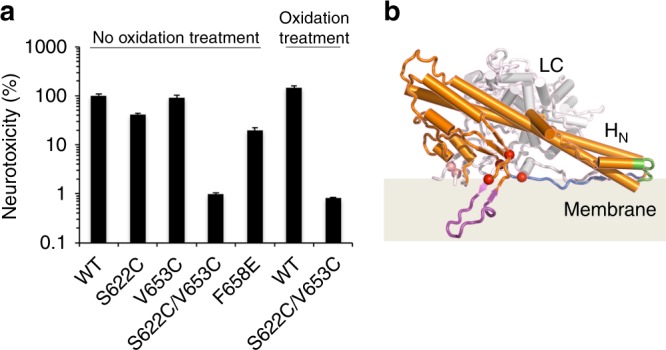
The BoNT-switch modulates neurotoxicity of BoNT/A1. **a** Neurotoxicity of BoNT/A1 variants was analyzed using the MPN assay. The paralytic half-times were determined and converted to the corresponding concentrations of wild-type BoNT/A1 using a concentration–response curve. The resulting toxicities were expressed relative to wild-type BoNT/A1 (*n* = 3–6, ± S.D.). **b** Proposed model for insertion of BoNT/A1 into lipid bilayer. The β2/β3 loop and the two putative membrane-interacting segments, F667–I685 and R827–R836, are colored in magenta, blue, and green, respectively. The inter-chain disulfide bond and four conserved carboxylates (D625, D629, E666, E670) are drawn as salmon and red spheres, respectively

To explore the functional relevance of the BoNT-switch to BoNT toxicity, we incorporated the S622C and V653C mutations into active BoNT/A1 (termed BoNT/A1^DS^). Since an endogenous disulfide linkage between LC and HC is crucial to LC translocation^55,56^, we could not directly measure the toxicity of BoNT/A1^DS^ under reducing conditions. Instead, we generated two single cysteine mutations (BoNT/A1^S622C^ and BoNT/A1^V653C^) as controls. We then examined the neurotoxicity of BoNT/A1 mutants at motor nerve terminals using ex vivo mouse phrenic nerve hemidiaphragm (MPN) assays^57^ (Fig. 5a). Remarkably, the naturally oxidized BoNT/A1^DS^ displayed a ~100-fold decreased toxicity, while BoNT/A1^V653C^ and BoNT/A1^S622C^ behaved like the WT toxin. When BoNT/A1^DS^ was further oxidized with copper phenanthroline, the decrease in neurotoxicity was ~180-fold. BoNT/A1^F658E^ has a ~5-fold decreased toxicity, which is likely due to the energetic costs of burying of an acidic residue in a hydrophobic pocket when tH_N_A transits into the acidic-pH conformation during LC translocation (Fig. 2b). These results prove that the structural change in the BoNT-switch as we observed in the crystal structure is essential to the extreme potency of BoNT/A1.

## Discussion

Like many other bacterial toxins and enveloped viruses, the capability of BoNT to trespass the endosomal membrane barrier is crucial for its function^22,58^. To achieve membrane insertion, these proteins commonly carry a buried hydrophobic segment as a molecular switch that is exposed only when the proteins sense the endosomal signals (e.g., acidic pH, enzymatic cleavage, membrane surface charges). Here, we identified such a molecular switch in BoNT/A1 that senses acidic environment and subsequently converts the water-soluble translocation domain into a membrane-competent state. We suggest that the highly conserved β2/β3 loop within the BoNT-switch may be the first part of H_N_ to insert into the membrane (Fig. 5b). This model is supported by the observation that two previously proposed membrane-interacting segments of H_N_A (segments F667–I685 and R827–R836) and the conserved disulfide linkage between LC and H_N_^56^ are close to the β2/β3 loop^33,34,43,44^. Furthermore, conserved carboxylates (D625, D629, E666, and E670) that were suggested to sense acidic pH and interact with the anionic lipid in their protonated forms^59^ are positioned at the base of the β2/β3 loop, which would favorably interact with a negatively charged membrane at acidic pH.

Our model of membrane binding would require an acidic pH-triggered structural re-orientation of BoNT/A1 to position H_N_A on the membrane surface. Such a structural rearrangement is possible because the flexible H_N_-H_C_ linker allows a 140^o^ rotation between H_N_ and H_C_ when BoNT/A1 is bound to the nontoxic nonhemagglutinin (NTNHA) protein at pH < 6.5 in the medium progenitor toxin complex (M-PTC)^60^. The driving force for the structural change likely involves acidic pH and the electrostatic attraction between the electropositive surface of BoNT/A1 with the negatively charged membrane^27^. Interestingly, the H_N_-H_C_ linker of TeNT is also modulated by pH, which may play a role in reorienting TeNT for the membrane insertion^61^. Furthermore, we speculate that the belt of BoNT/A1 may modulate the conformational change of H_N_A by interacting with and shielding the hydrophobic surface of the BoNT-switch. The belt is likely unfolded upon membrane binding and vesicle acidification that may help to release the BoNT-switch.

It is well known that some viral-fusion proteins, such as influenza virus hemagglutinin, vesicular stomatitis virus GP, and Ebola virus GP, contain a hydrophobic loop that is buried at neutral pH, but released at acidic pH to insert the fusion protein into the lipid bilayer^3,46^. Our complementary structural and functional studies reveal a surprising similarity between the β2/β3 loop of the BoNT-switch, which is conserved in almost all known BoNTs, and the IFL of Ebola virus GP2. Moreover, H_N_A carries a pair of long α-helices that resemble the coiled-coil viral-fusion proteins (e.g., influenza hemagglutinin and HIV-1 gp41)^10^. These viral proteins undergo dramatic structural reorganization of these helices after the fusion loop penetrates the membrane^3,62,63^. We speculate that the formation of protein-conducting channel of BoNT may similarly involve a large reorganization of the long helices within tH_N_A.

Interestingly, the β2/β3 loop of BoNT is situated at the middle of the long helices that likely break down into shorter helical segments following membrane insertion. In this scenario, our model may represent the first step of membrane insertion, in which the β2/β3 loop brings the long helices of H_N_A closer to the vesicle membrane and promotes its reorganization to form the translocation channel. Another possibility is that the β2/β3 loop may constitute part of the transmembrane hairpin and H_N_A may form β-hairpin channels reminiscent of anthrax toxin^22^. Formation of a β-hairpin channel requires oligomerization of BoNT, which is also consistent with our observation that acidic pH tends to promote BoNT/A1 oligomerization on liposomes, which is partly regulated by the BoNT-switch. It is worth noting that our data argues that the mechanism of BoNT membrane insertion is different from some other bacterial toxins, such as diphtheria toxin and colicin, which also have an α-helical translocation domain similar to BoNT. Those toxins do not have a viral-fusion-peptide-like switch, and they insert into membranes through exposure of a helical hairpin without a significant change in secondary structures^64^.

In summary, our studies demonstrate that BoNT/A1 contains a molecular switch that regulates channel formation and LC delivery, so it only happens in the right cellular location at the right time. The importance of the BoNT-switch to the neurotoxicity of BoNT/A1 suggests that blocking the conformational transition with an antibody or small molecule inhibitors holds promise as an alternative strategy to neutralize these extremely lethal toxins. This is also supported by a report that antibodies from the sera of some cervical dystonia patients with immune-resistance to BoNT/A1 bound to a region of BoNT/A1 (S659–G677) that is within the BoNT-switch^65^. These unique human antibodies are worthy of further characterization. Because of its functional importance and high sequence conservation among all known BoNTs, the BoNT-switch could be an ideal target for the development of the long sought-after cross-serotype therapeutic intervention for botulism.

## Methods

### Statistical method

No statistical method was used to predetermine sample size. The experiments were not randomized and were not performed with blinding to the conditions of the experiments.

### Construct design and cloning

The sequences corresponding to full-length BoNT/A1 carrying two substitutions at the active site (R363A/Y366F) (BoNT/A1i), and tH_N_A (residues K547–K871) from BoNT/A1-producing *C. botulinum* strain 62 A were cloned into expression vectors pGEX-6p-1 and pCDFduet-1, respectively. ciA-D12 was cloned into pGEX-6p-1. Sequences of PCR primers are provided in Supplementary Table 1. To facilitate protein purification, a His_6_-tag followed by a PreScission cleavage site was introduced to the N-terminus of tH_N_A. BoNT/A1i and ciA-D12 were produced with an N-terminal GST-tag followed by a PreScission cleavage site. All tH_N_A, BoNT/A1, BoNT/A1i, and ciA-D12 mutations were generated by QuikChange site-directed mutagenesis (Agilent).

### Protein expression and purification

All recombinant proteins were expressed in the *E. coli* strain BL21-RIL (DE3) (Novagen). Bacteria expressing BoNT/A1i and ciA-D12 were grown at 37 °C in LB medium in the presence of the appropriate selecting antibiotics. Expression was induced with 1 mM isopropyl-b-D-thiogalactopyranoside (IPTG) when OD_600_ reached 0.6. The temperature was then decreased to 16^o^C and expression was continued for 16 h. For the expression of tH_N_A, bacteria were grown at 37 °C in TB medium and expression was induced with 1 mM IPTG when OD_600_ reached 0.9. The temperature was then decreased to 19 °C and expression was continued for 16 h. The cells were harvested by centrifugation and stored at −20 °C until use.

For protein purification, bacteria were resuspended in Tris buffer (50 mM Tris pH 8.0, 400 mM NaCl) with 0.4 mM PMSF and lysed by sonication. His_6_-tagged proteins (tH_N_A wild type and mutants) were purified using a Ni-NTA (nitrilotriacetic acid, Qiagen) affinity column in a buffer containing 50 mM Tris (pH 8.0), 400 mM NaCl, and 10 mM imidazole and subsequently eluted in the same buffer containing 150 mM imidazole. The eluted fractions of each protein were pooled and dialyzed overnight at 4 °C against a buffer composed of 20 mM Hepes (pH 8.0) and 50 mM NaCl, followed by His-tag removal using PreScission protease. The protein was further purified by MonoQ ion-exchange chromatography (GE Healthcare) in a buffer containing 20 mM Hepes (pH 8.0) and eluted with a NaCl gradient, followed by Superdex-75 size-exclusion chromatography (SEC; GE Healthcare, Germany) in 10 mM Hepes (pH 7.0) and 150 mM NaCl. GST-tagged BoNT/A1i and the ciA-D12 were purified using Glutathione Sepharose 4B resins (GE Healthcare) in 50 mM Tris (pH 8.0), 400 mM NaCl, and eluted from the resins after on-column cleavage using PreScission protease. The protein was further purified by Superdex-200 Increase or Superdex-75 SEC in 10 mM Hepes (pH 7.0) and 150 mM NaCl for BoNT/A1i and ciA-D12, respectively. Each protein was concentrated to ~5 mg/ml using Amicon Ultra centrifugal filters (Millipore) and stored at −80 °C until used for further characterization or crystallization.

Wild type and mutated recombinant full-length activated BoNT/A1 were produced under biosafety level 2 containment (project number GAA A/Z 40654/3/123) recombinantly in K12 *E. coli* strain in Dr. Rummel’s lab^66^. BoNT/A1 variants carrying C-terminal His_6_-tag were purified on Co^2+^-Talon matrix (Takara Bio Europe S.A.S., France) and eluted with 50 mM Tris-HCl (pH 8.0), 150 mM NaCl, and 250 mM imidazole. For proteolytic activation, BoNT/A1 was incubated for 16 h at room temperature with 0.01 U bovine thrombin (Sigma-Aldrich Chemie GmbH, Germany) per µg of BoNT/A1. Subsequent gel filtration (Superdex-200 SEC; GE Healthcare, Germany) was performed in phosphate buffered saline (pH 7.4).

The purified tH_N_A^DS^ and BoNT/A1^DS^ were oxidized by copper 1,10-phenanthroline in vitro. Oxidation of the tH_N_A^DS^ mutant was confirmed by gel-shift analysis (Supplementary Fig. 7b).

The purified ciA-D12 (S124C) was labeled with Alexa Fluor C_2_ 647 maleimide (Molecular Probes) according to the manufacturer’s instructions. The labeled ciA-D12 was further purified by MonoQ ion-exchange chromatography in 10 mM Hepes (pH 8.0) and eluted with a NaCl gradient. The calculated dye to protein ratio was ~1 mole of dye per mole of ciA-D12. BoNT/A1i-ciA-D12 complex were prepared by mixing BoNT/A1i and ciA-D12 in 1:1.5 molar ratio and the complex was purified by size-exclusion chromatography using Superdex-200.

### Crystallization

Initial crystallization screens were performed using a Phoenix crystallization robot (Art Robbins Instruments) and high-throughput crystallization screen kits (Hampton Research), followed by extensive manual optimization. The best single crystals of tH_N_A were grown at 18 °C by the sitting-drop vapor diffusion method. The protein at a concentration of 5 mg/ml was mixed in a 2:1 (v/v) ratio with a reservoir solution containing 1.4 M sodium potassium phosphate (pH 5.1) and 0.1 M potassium sodium tartrate tetrahydrate. Crystals of tH_N_A^F658E^ were grown with a protein concentration at 3 mg/ml at 18 °C with a reservoir solution containing 2.4 M ammonium phosphate, 0.1 M Tris (pH 8.5), and 0.05 % n-dodecyl β-D-maltoside. The crystals of tH_N_A were cryoprotected in the original mother liquor supplemented with 30% (v/v) ethylene glycol. The crystals were dehydrated by air dry for 5 minutes and then frozen in liquid nitrogen. The crystals of tH_N_A^F658E^ were cryoprotected with 2.5 M lithium sulfate.

Data collection and structure determination: After screening numerous crystals at the Stanford Synchrotron Radiation Lightsource (SSRL) or Advanced Photon Source (APS), the best X-ray diffraction datasets for tH_N_A and tH_N_A^F658E^ crystals were collected at 100 K at beam line BL9-2, SSRL. The data were processed with iMOSFLM^67^. Data collection statistics are summarized in Table 1. The structure of tH_N_A or tH_N_A^F658E^ was determined by molecular replacement using the Phaser software^68^ with tH_N_A of BoNT/A1 as the search model (PDB code 3V0A)^60^. Manual model building and refinement was performed in COOT^69^, PHENIX^70^, and CCP4 suite^71^ in an iterative manner. The refinement progress was monitored with the free R value using a 5% randomly selected test set^72^. The structures were validated through the MolProbity^73^ and showed excellent stereochemistry. Structural refinement statistics are listed in Table 1. Residues 858–871 of chain A and residue 871 of chain B of tH_N_A and residues 647–649 of tH_N_A^F658E^ are invisible in the electron density map that likely indicates local flexibility. One and nine phosphate molecules were identified in the crystal structures of tH_N_A and tH_N_A^F658E^, respectively, that could be attributed to the high phosphate concentration in the corresponding crystallization buffer. All structure figures were prepared with PyMol (http://www.pymol.org).

### 8-anilinonaphthalene-1-sulfonic acid binding assay

The wild type or mutant tH_N_A was incubated at 0.1 mg/ml (~2.68 μM) with 100 μM ANS for ten minutes in either 50 mM sodium acetate (pH 4.0–5.6), 50 mM sodium cacodylate (pH 6.2), or 50 mM Hepes (pH 7.0). All buffers contained 100 mM NaCl. Fluorescence spectra were recorded at 25 °C in a Molecular Devices SpectraMax M2e spectrophotometer with excitation at 370 nm. The emission spectrum was collected from 420 to 640 nm. The fluorescence intensity was corrected by subtraction of background fluorescence from ANS in buffer lacking protein. Fluorescence emission was calculated as the area under the spectra. Error bars indicate SD of three replicate measurements.

### Thermal denaturation assay

The thermal stability of wild type or mutant tH_N_A was measured using a fluorescence-based thermal shift assay on a StepOne real-time PCR machine (Life Technologies). Immediately before the experiment, the protein (2.7 μM) was mixed with the fluorescent dye SYPRO Orange (Sigma-Aldrich) at pH 7.0 or pH 4.6. The samples were heated from 25 °C to 90 °C in ~45 min. The midpoint of the protein-melting curve (Tm) was determined using the analysis software provided by the instrument manufacturer. The data obtained from three independent experiments were averaged to generate the bar graph. The Tm of tH_N_A wild type and reduced tH_N_A^DS^ at pH 4.6 is not determined due to high fluorescence signal at starting temperature.

### Analytical size-exclusion chromatography

tH_N_A at protein concentrations ranging from 0.1 to 1 mg/ml (~2.7–27 μM) were incubated at 4 °C for 30 minutes in various buffers that contained 0.1 M NaCl and either 50 mM sodium acetate (pH 4.4–5.6) or 50 mM Hepes (pH 7.4). BoNT/A1i at 4 mg/ml (27 μM) was incubated in buffers at pH 4.4 or pH 7.4. Reduced tH_N_A^DS^ was prepared by pre-incubation with 5 mM TCEP. Protein oligomerization was resolved using Superdex-200 SEC.

### Liposome co-sedimentation assays

Liposomes were prepared by extrusion method using Avanti Mini Extruder according to manufacturer’s protocol. Briefly, lipids (Avanti Polar Lipid) were dissolved in chloroform while GT1b trisodium salt (Santa Cruz Biotechnology) was dissolved in methanol. The lipids at the indicated molar ratios were mixed and then dried under nitrogen gas and placed under vacuum for at least 2 h. The dried lipids were rehydrated and were subjected to five rounds of freezing and thawing cycles. Unilamellar vesicles were prepared by extrusion through a 100 nm pore membrane using an Avanti Mini Extruder according to the manufacturer’s instructions.

To study the pH-dependence of tH_N_A–lipid association, 0.2 μM protein was incubated with 0.15 mg/ml asolectin liposomes (Sigma-Aldrich) in 150 mM NaCl with either 20 mM sodium acetate (pH 4.4–5.0) or 20 mM Hepes (pH 7.5) at room temperature for 2 h. The samples were centrifuged at 160,000× *g* for 40 min. The supernatant was removed and the pellets were resuspended twice in the same buffer and were re-centrifuged. Liposome-bound proteins were analyzed by comparing the supernatant and pellet fractions on SDS-PAGE.

Effect of lipid composition on tH_N_A–lipid binding was studied by mixing 0.27–2.7 μM of tH_N_A with 0.1 mg/ml liposome containing either 60% L-α-phosphatidylcholine (PC), 40% cholesterol; 60% L-α-phosphatidylserine (PS), 40% cholesterol; or 30% PC, 30% PS, 40% cholesterol (Encapsular Nano Sciences). The protein–liposome mixture was incubated in 0.1 M NaCl, 50 mM sodium acetate (pH 4.4) at room temperature for 2 h and centrifuged.

The effect of disulfide formation in the BoNT-switch on tH_N_A–lipid binding was studied by a low-centrifugal field protocol as described using vesicles containing 1-oleoyl-2-(9,10-dibromostearoyl)phosphatidylcholine (OBPC)^74^. OBPC has density of 1.2 g/cm^3^ and sediments easily in relatively low centrifugal field^75^. A volume of 0.2 μM tH_N_A or mutants were incubated with liposomes composed of 80% OBPC and 20% DOPS in 0.1 M NaCl, 50 mM sodium acetate (pH 4.6), 1 mM KCl, and 1 mM EDTA at room temperature for 2 h. The samples were spun progressively at 4000×, 9000×, and 16,000× *g* for 30 min each. Supernatant and pellet were separated and analyzed by SDS-PAGE.

### Calcein dye release assay

Dried lipid (20 mg/ml) was resuspended in 150 mM NaCl, 20 mM Hepes (pH 7.0), 1 mM EDTA, 50 mM calcein. Free calcein dye was separated from calcein-entrapped liposomes by desalting (Zeba). Fluorescence was measured on a Spectramax M2e cuvette module with excitation at 493 nm and emission at 525 nm. In the assay, liposomes were diluted either in 150 mM NaCl, 20 mM sodium acetate (pH 4.6), 1 mM EDTA, or in 150 mM NaCl, 20 mM Hepes (pH 7.0), 1 mM EDTA to give a final concentration of 0.2 mM and incubated until the fluorescence signal was stable. Wild type or mutant tH_N_A was added at 0.2 μM and the fluorescence intensity was recorded for 10 minutes. The reaction was stopped by adding 0.1% Trion X100. The percentage of fluorescence change was calculated as the ((F − F_initial_) /  F_final_).

### Membrane depolarization assay

Depolarization was measured as previously described with some modifications^36^. Briefly, liposomes composed of 70% DOPC, 20% DOPS, 10% cholesterol were prepared in 200 mM NaCl, 1 mM KCl, 10 mM Hepes (pH 7.0). To create a trans–positive membrane potential ( + 135 mV), liposomes were diluted in 200 mM KCl, 1 mM NaCl with either 10 mM sodium acetate (pH 4.6–5.8) or 10 mM Hepes (pH 7.0). Membrane potential was monitored using 6 μM ANS. ANS is sensitive to membrane potential and has been used as a measure of the magnitude of the diffusion potential^37^. ANS is strongly fluorescent when bound to lipids and gives an additional increase in fluorescence intensity when a trans–positive potential is present. Valinomycin was added at time 0-second to give a final concentration of 30 nM. At 180-second, wild type, or mutant tH_N_A were added at 0.2 μM and the fluorescence intensity at 490 nm was monitored for 3 minutes with excitation at 380 nm. The experiments were repeated three times independently.

### Mouse phrenic nerve hemidiaphragm assay

The MPN assay was performed employing 20–30 g NMRI mice (Janvier SA, France) as described previously^19,57^. According to §4 Abs. 3 (killing of animals for scientific purposes, German animal protection law (TSchG)), animals were sacrificed by trained personnel before dissection of organs and its number reported yearly to the animal welfare officer of the Central Animal Laboratory and to the local authority, Veterinäramt Hannover. First, mice were euthanized by CO_2_ anesthesia and subsequent exsanguination via an incision of the ventral aspect of the throat. The chest of the cadaver was opened, the phrenic nerve hemidiaphragm tissue was explanted and placed into an organ bath. The phrenic nerve was continuously stimulated at 5–25 mA with a frequency of 1 Hz and with a 0.1 ms pulse duration. Isometric contractions were transformed using a force transducer and recorded with VitroDat Online software (FMI GmbH, Germany). The time required to decrease the amplitude to 50% of the starting value (paralytic half-time) was determined. To allow comparison of the altered neurotoxicity of mutants with BoNT/A1 wild type, a power function (y(BoNTA1; 0.75, 1.5, 3.0, 6.0 pM) = 98.536 × ^−0.267^, *R*^2^ = 0.9693) was fitted to a concentration–response curve consisting of four concentrations determined in 3–5 technical replicates. Resulting paralytic half-times of the BoNTA1 mutants were converted to concentrations of the wild type employing the above power functions and finally expressed as relative neurotoxicity.

### Single-molecule analysis of oligomerization

ciA-D12 construct was kindly provided by Dr. Charles B. Shoemaker^54^. ciA-D12 contains two buried cysteines that are inaccessible for labeling. An additional cysteine (S124C) was introduced into ciA-D12 through site-directed mutagenesis. Purified ciA-D12 was labeled with Alexa-647 maleimide (Thermo Fisher Scientific) and subsequently purified with cation exchange. The labeling efficiency was determined to be over 99% using UV-Vis spectroscopy. BoNT/A1i was expressed and purified as described above. The complexes with D12* were prepared by incubating with a 1.5-fold excess of D12*. Free D12* was removed by Superdex-200 SEC.

Liposomes containing 10% GT1b, 69% DOPC, 20% DOPS, 0.5% rhodamine-PE, and 0.5% biotin-PE were prepared by extrusion through a 100 nm pore membrane. To form proteoliposomes, 10 nM BoNT/A1i–D12* or oxidized BoNT/A1i^DS^–D12* was incubated with 0.5 mg/ml lipid at room temperature for 1 h at the pH indicated. The mixture was then diluted 1000-fold and incubated for 5 minutes in a passivated, quartz microscope chamber functionalized with streptavidin. The biotinylated liposomes were retained and unbound proteins are washed away by exhaustive rinsing with buffer. At the low densities needed for optical resolution of individual liposomes, we could observe sufficient liposomes for statistical analysis, while minimizing the probability that a diffraction-limited spot would contain multiple liposomes. Samples were imaged using a prism-based Total Internal Reflection Fluorescence (TIRF) microscope. Samples were first excited with a laser diode at 640 nm (Newport Corporation, Irvine, CA) to photobleach Alexa 647-labeled BoNT/A1i–D12* or BoNT/A1i^DS^-D12* molecules followed by excitation with a diode pumped solid state laser at 532 nm (Newport Corporation, Irvine, CA) to probe for Rhodamine-labeled liposomes. Emission from protein and lipids was separated using an Optosplit ratiometric image splitter (Cairn Research Ltd., Faversham UK) containing a 645 nm dichroic mirror, a 585/70 band pass filter for Rhodamine, and a 670/30 band pass filter for Alexa 647 (all Chroma, Bellows Falls, VT). The replicate images were relayed to a single iXon EMCCD camera (Andor Technologies, Belfast, UK) at a frame rate of 10 Hz. Data were processed in MATLAB to cross-correlate the replicate images and extract time traces for diffraction-limited spots with intensity above baseline. Single-molecule traces were hand selected based on the exhibition of single-step decays to baseline during 640 illumination and the appearance of Rhodamine emission during 532 illumination.

### SAXS data collection and analysis

Purified tH_N_A was exchanged into a buffer containing 200 mM NaCl, 5 mM DTT, 0.1 mM EDTA, 55 mM Hepes (pH 7.0), and the protein samples were concentrated to 10 mg/ml. SEC-SAXS experiment was performed using Bio-SAXS beam line BL4-2 at Stanford Synchrotron Radiation Lightsource (SSRL) in a similar manner to our previous report^76^. Experimental setup and structural parameters are summarized in Supplementary Table 2. Briefly, the program SasTool (http://ssrl.slac.stanford.edu/~saxs/analysis/sastool.htm) was employed for data reduction to generate background-subtracted scattering profiles. The script fplcplots, implemented in the program AUTORG^77^, was used for consecutive Guinier analysis and assessing data quality. Sample was eluted as a sharp peak and no significant radiation damage or concentration dependence was observed during the elution. The theoretical scattering curve from an atomic structure (tH_N_A of PDB code 3BTA) was computed and was fitted with experimental data using the program CRYSOL^78^. Ab initio modeling was performed using the program DAMMIF^79^ and the averaged and filtered model from 20 independent ab initio models was generated by the program DAMAVER^80^.

## Electronic supplementary material

Supplementary Information

Reporting Summary

## Data Availability

Atomic coordinates and structure factors for tH_N_A and tH_N_A^F658E^ have been deposited in the Protein Data Bank under accession codes 6DKK [10.2210/pdb6DKK/pdb] and 6 MHJ [10.2210/pdb6MHJ/pdb], respectively. All relevant data are available within the paper and its Supplementary Information files.
