# Supplementary material for: A viral-fusion-peptide-like molecular switch drives membrane insertion of botulinum neurotoxin A1
Source: Nat Commun. 2018 Dec 18;9:5367. doi: 10.1038/s41467-018-07789-4 (PMC6299077; doi:10.1038/s41467-018-07789-4)
Supplement: Supplementary file 1 — Supplementary Information [file 41467_2018_7789_MOESM1_ESM.pdf]

A viral-fusion-peptide-like molecular switch drives membrane insertion of botulinum neurotoxin A1

K. Lam et al.

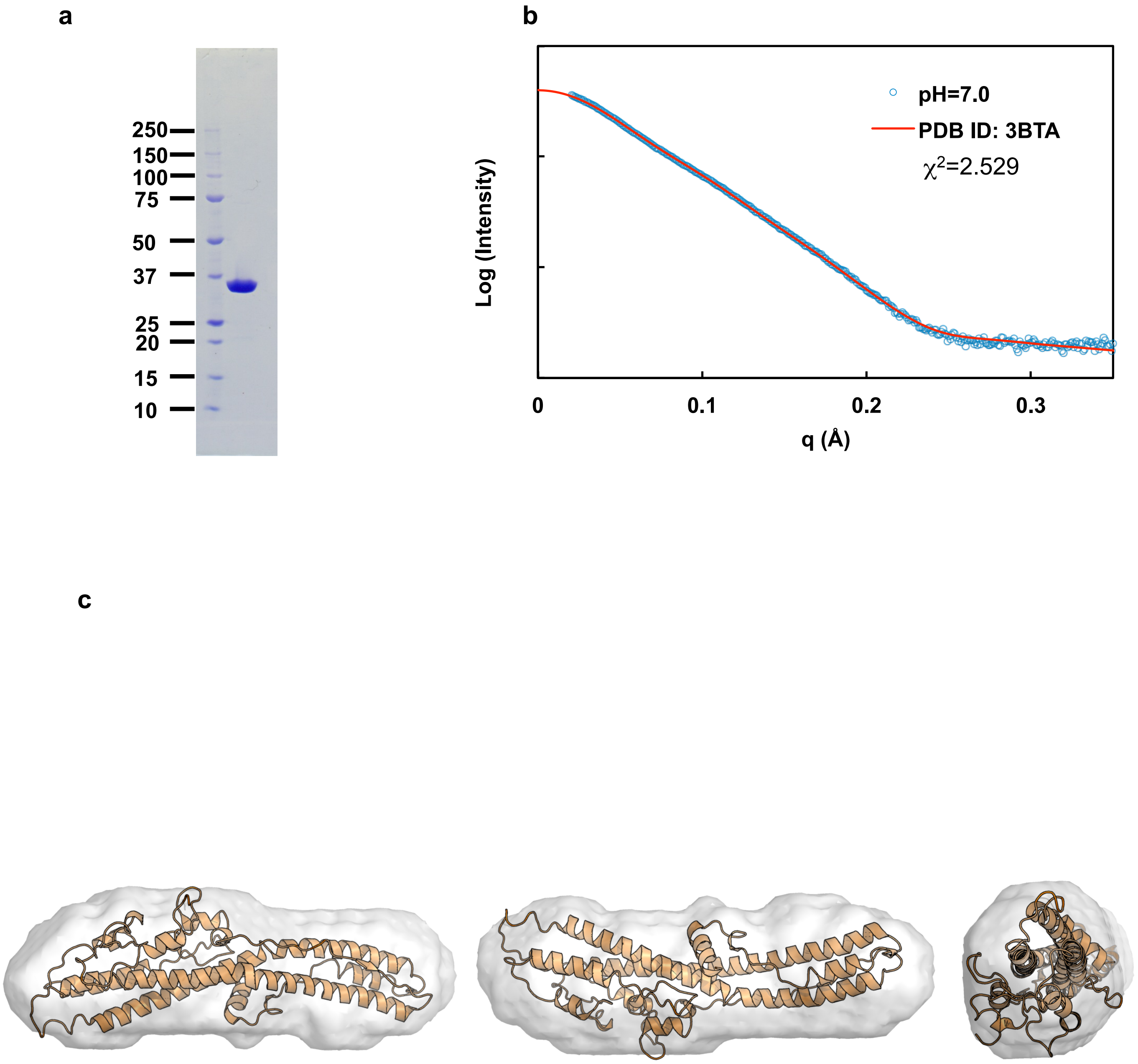

Supplementary Figure 1

SEC-SAXS studies of tH_NA .

(**a**) The purity of tH_NA was analyzed by SDS-PAGE and Coomassie Blue staining. (**b**) SAXS profile of the tH_NA at pH 7.0 and curve fitting with tH_NA structure (extracted from PDB code 3BTA). (**c**) Ab initio model of the solution structure of tH_NA. The molecular envelope is compared to the crystal structure.

a

Residues	predicted pKa value
**E620**	**5.2**
D625	3.92
**D629**	**5.88**
D650	3.65
D651	4.31
**E666**	**6.85**

b c

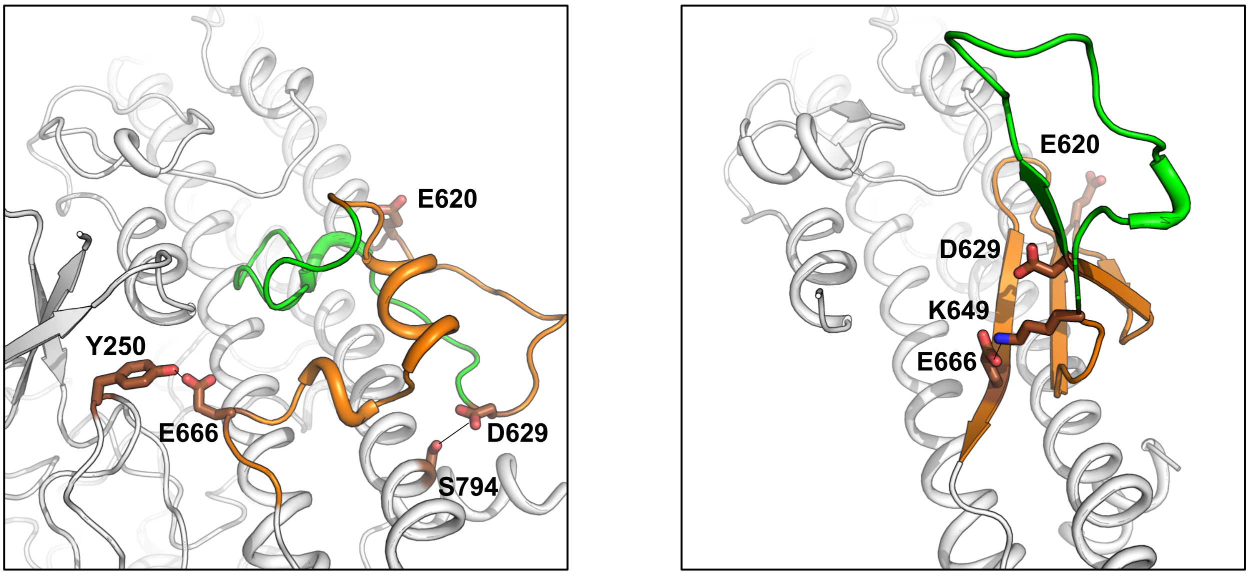

Supplementary Figure 2

Titratable charged residues in the BoNT-switch.

(**a**) The pKa values of the carboxylic acid residues of BoNT-switch were calculated by Propka3.0[1]. Three carboxylates that show increased pKa values are highlighted. (**b, c**) Interactions of D629 and E666 with other polar residues are very different in BoNT/A (PDB code: 3BTA) (**b**) and the acidic pH conformation of tH_NA (**c**). The carboxylates and the interacting residues are drawn as brown sticks.

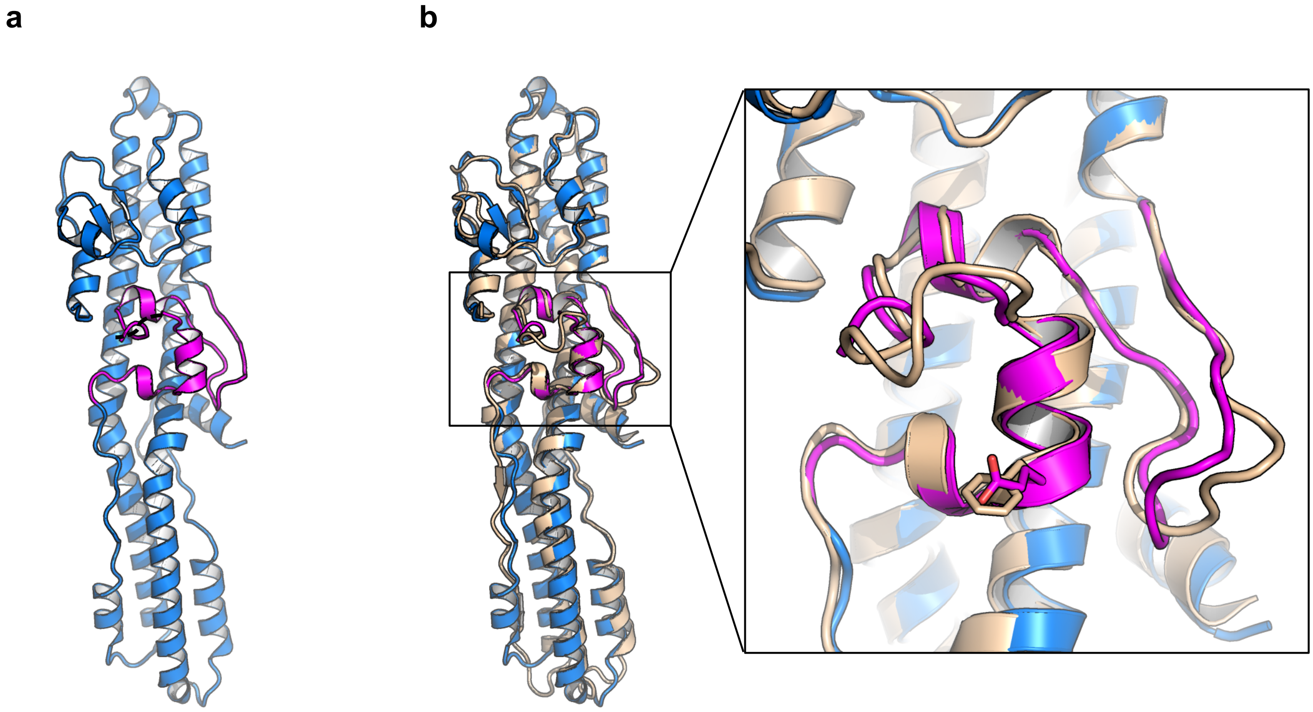

Supplementary Figure 3

Structure of tH_NA^{F658E}. (**a**) The BoNT-switch is highlighted in magenta. Three residues (L647–K649) that do not have visible electron density are marked as dotted line.
(**b**) Superposition of tH_NA^{F658E} with the corresponding region that was extracted from the structure of full length BoNT/A (PDB code: 3BTA). (Boxed) A close-up view of the BoNT-switch. The mutated residue (F658E) is drawn as sticks.

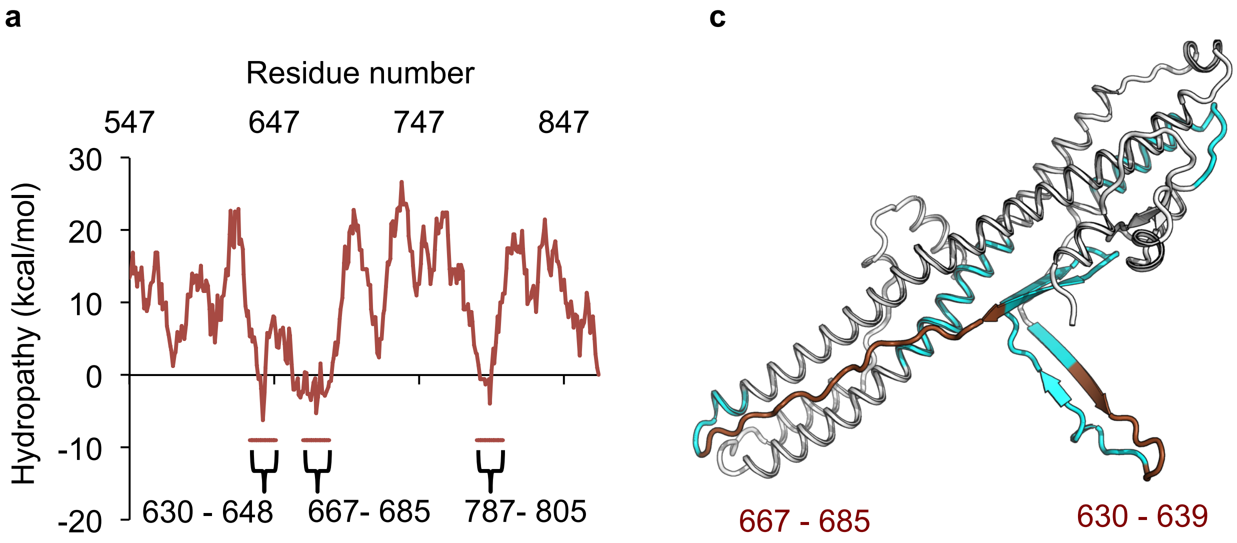

b

Algorithm	TM segments
MPEx	630 – 648, 667 – 685, 787 – 805
TMPred	627 – 648, 652 – 676, 671 – 690, 787 – 805
DAS	630 – 639, 654 – 686
TMAP	626 – 645, 657 – 685
MOMENT	596 – 615, 626 – 648, 649 – 692

Supplementary Figure 4

Prediction of the transmembrane segments (TM) of tH_NA by different computational programs. (**a**) Hydropathy plot for the tH_NA according to Wimley-White Scale[2]. (**b**) A summary of TM segments predicted by MPEx[3], TMPred[4], DAS[5], TMAP[6], and MOMENT[7]. (**c**) The putative TM segments are highlighted (cyan) in tH_NA. Two fragments of tH_NA (residues 630-639 and 667-685) that are identified by all computational programs are colored in brown.

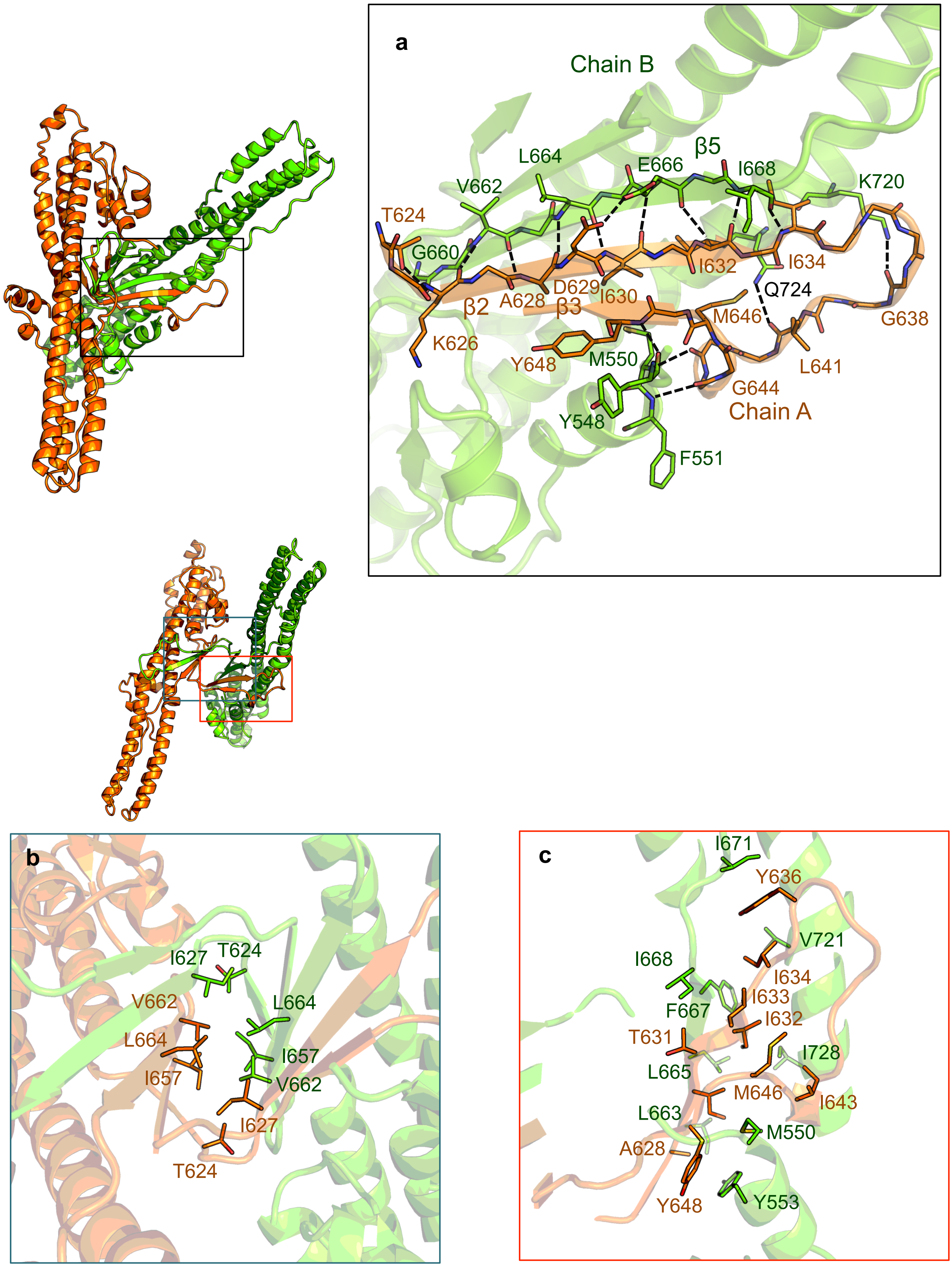

Supplementary Figure 5

Interactions between tH$_{N}$A dimer.

(**a**) The β2/β3 hairpin of chain A (sticks, orange) forms extensive hydrogen bonds (dotted lines) with β5 (sticks, green) and the N-terminal helix of chain B. The side chain of non-interacting residues are omitted for clarity. (**b, c**) Hydrophobic interactions between chain A and chain B. The residues participated in the interaction are shown as sticks and labeled.

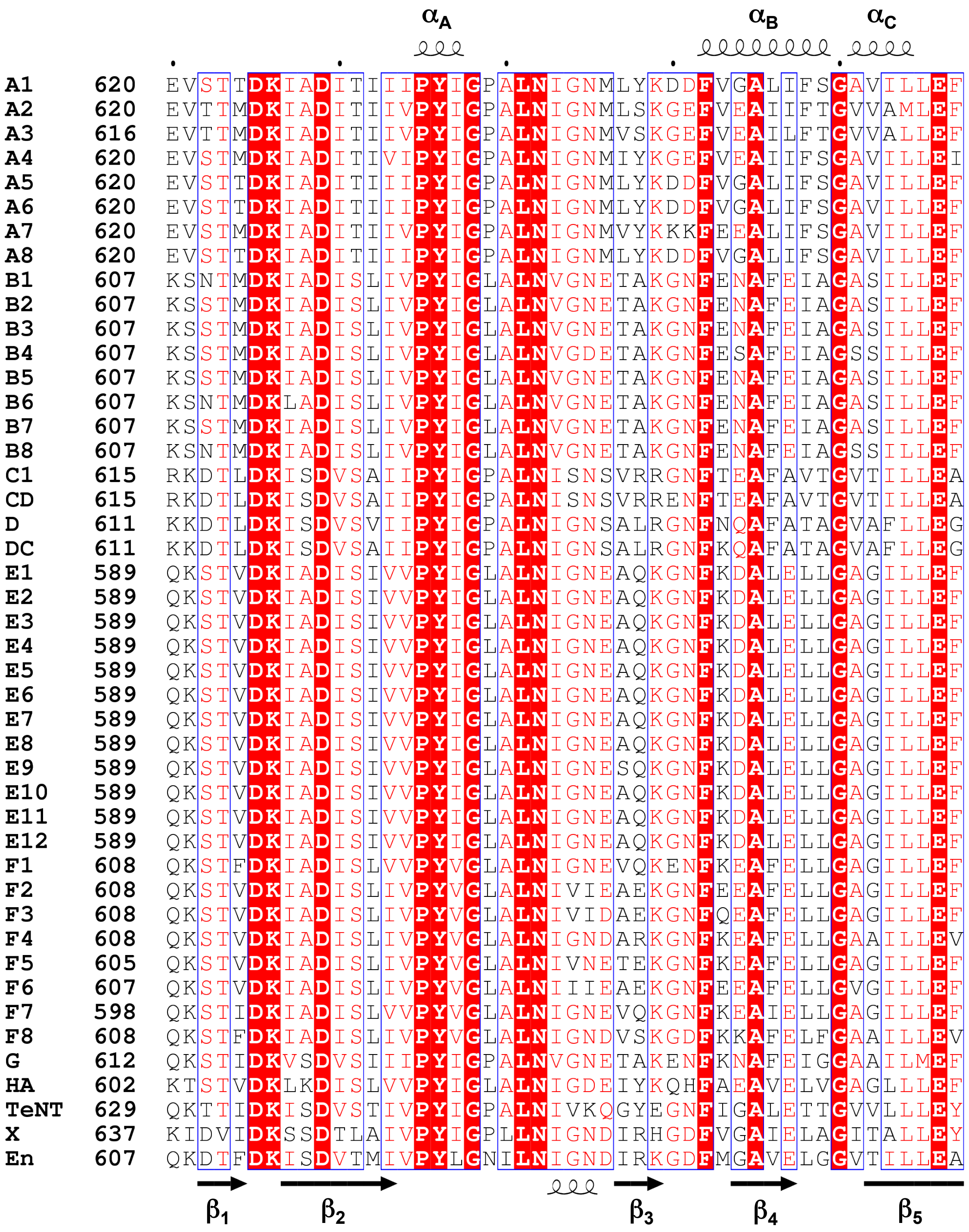

Supplementary Figure 6

Sequence alignments of the BoNT-switch region (E620–F667, BoNT/A1 numbering) from 44 BoNT subtypes and TeNT.

Sequence alignments were made using Clustal Omega and ESPript 3.0. The secondary structures of the BoNT-switch corresponding to the neutral or the acidic pH conformations are shown above and below the aligned sequences, respectively.

a

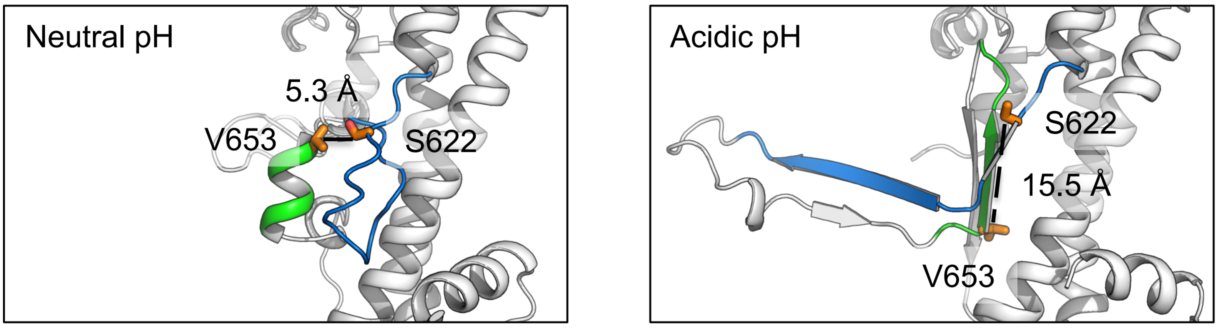

b

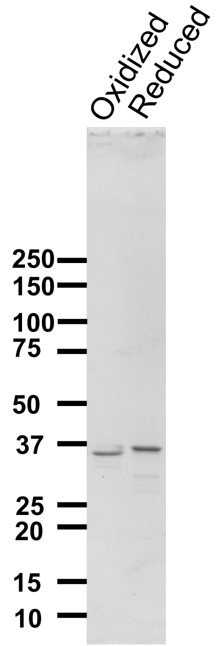

Supplementary Figure 7

(**a**) The distance between residues S622 and V653 (orange) on the α_B (green) and $loop_A$ (blue) is ~5.3 Å at a neutral pH (extracted from 3BTA) and ~15.5 Å at an acidic pH. (**b**) Treating tH_NA^{DS} by Cu^{2+} phenanthroline led to a downward shift of protein band in SDS-PAGE.

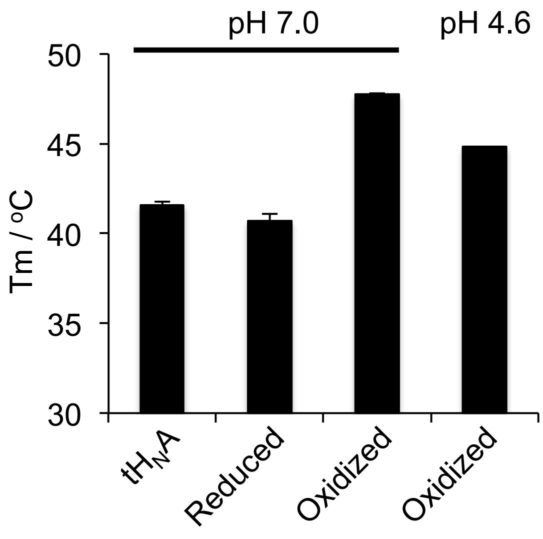

Supplementary Figure 8

Thermal stability of tH_NA and tH_NA^{DS} at neutral and acidic pH.

The thermal stability of tH_NA and tH_NA^{DS} (oxidized or reduced) were measured using a fluorescence-based thermal shift assay on a StepOne real-time PCR system (ThermoFisher). Protein melting was monitored using a hydrophobic dye, SYPRO Orange (Sigma-Aldrich), as the temperature was increased in a linear ramp from 20 °C to 95 °C. The midpoint of the protein-melting curve (Tm) was determined using the software provided by the instrument manufacturer. The data are presented as mean ± S.D., n = 3. The Tm of wild type tH_NA and reduced tH_NA^{DS} at pH 4.6 could not be determined due to significant protein unfolding at the starting temperature.

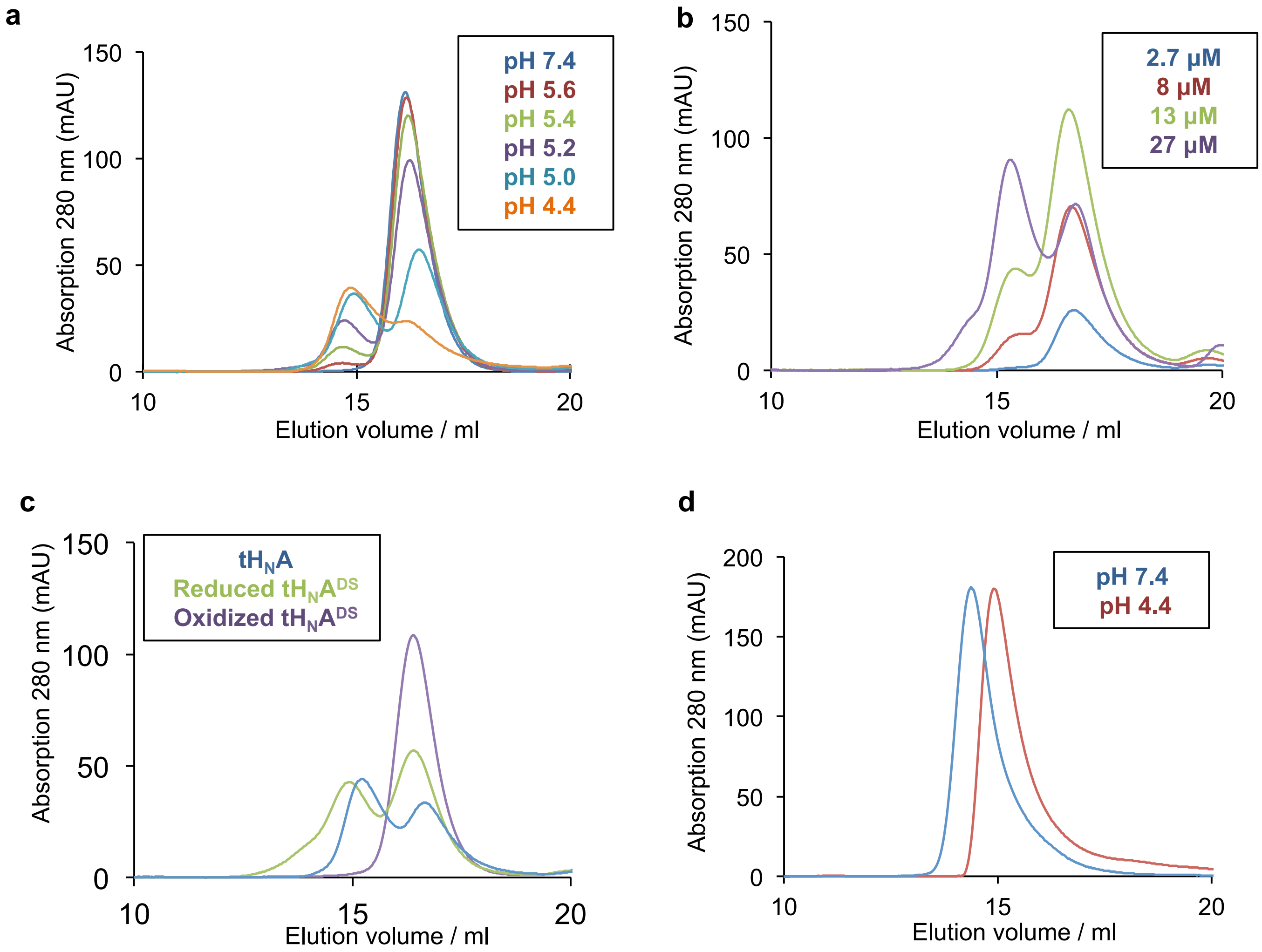

Supplementary Figure 9

Protein aggregation in solution was analyzed by Superdex S200 chromatography. tH_NA self-association was examined (**a**) at various pH (at ~27 µM) and (**b**) at various protein concentrations (at pH 4.4). (**c**) The effect of oxidation on the oligomerization of tH_NA^{DS}. (**d**) Gel filtration analysis of BoNT/A1i in buffer of at pH 7.4 or pH 4.4.

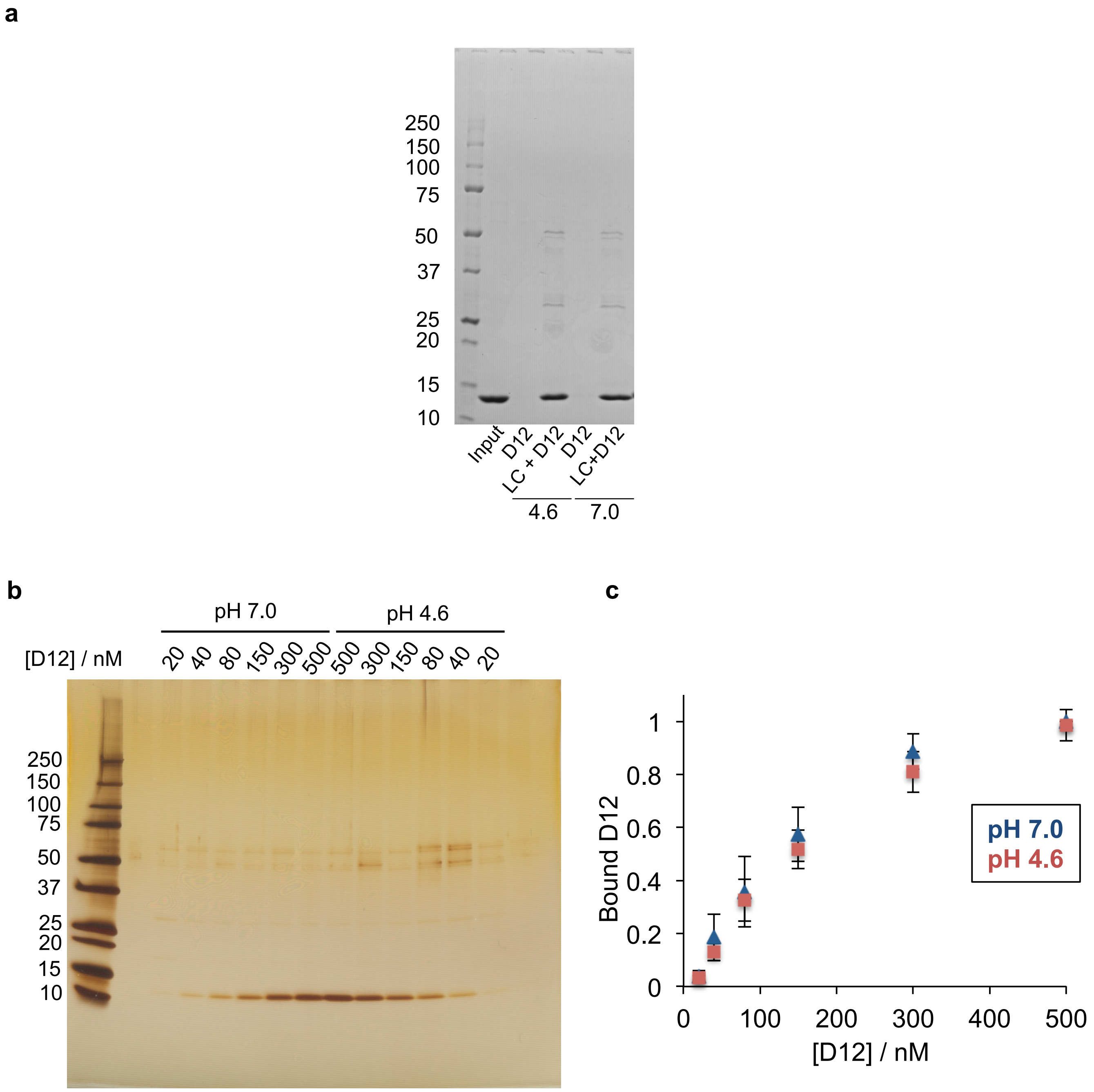

Supplementary Figure 10

LC/A-ciA-D12 interactions are not affected by pH.

(**a**) LC/A was covalently immobilized on CNBr beads as bait by amine crosslinking. ciA-D12 was incubated with immobilized LC/A at a 1.5 : 1 molar ratio at pH 4.6 and pH 7.0. After washing with the same buffer, the pull-downed ciA-D12 was denatured with SDS loading dye and detected by SDS-PAGE analysis. (**b**) Immobilized LC/A was incubated with various concentration of ciA-D12 (20–500 nM) at pH 4.6 and pH 7.0. The gel was stained by silver staining. (**c**) The band intensities of the bound ciA-D12 were quantified and represented as (Intensity / Intensity of maximal binding). The mean values of the relative band intensities are shown and the error bar represents standard deviation of three independent experiments.

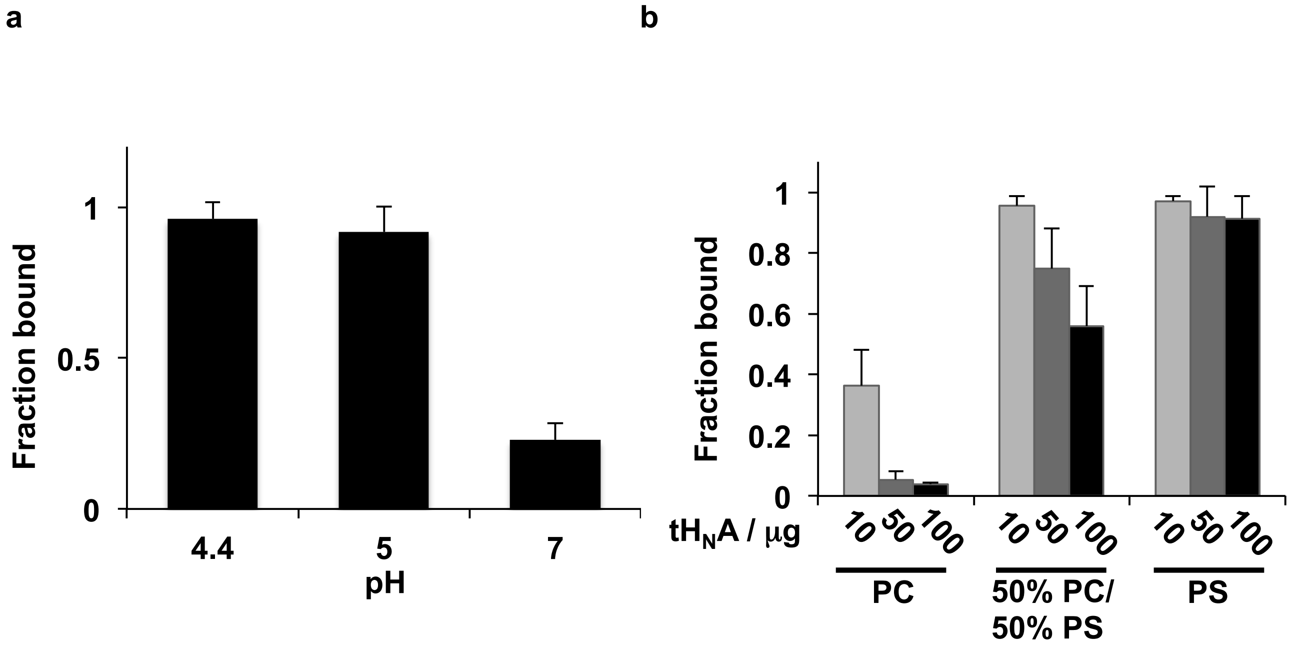

Supplementary Figure 11

Quantification of results shown in Figure 1b (**a**) and 1c (**b**). The intensities of protein bands were quantified for the input and the supernatant fractions. The faction of the bound-tH_NA was calculated as 1 - (band intensity of unbound protein / input protein). The data are presented as mean ± S.D., n = 3.

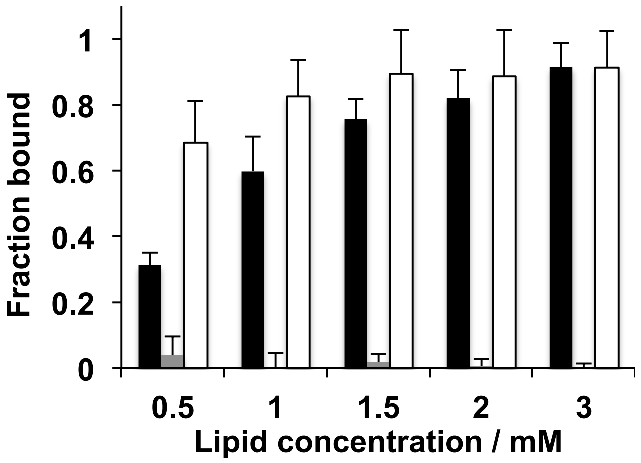

Supplementary Figure 12

Quantification of the results shown in Figure 3b. The intensities of protein bands were quantified for the input and the supernatant fractions. The faction of the bound-tH_NA was calculated as 1 - (band intensity of unbound protein / input protein). The data are presented as mean ± S.D., n = 3.

Figure 1c, row 1

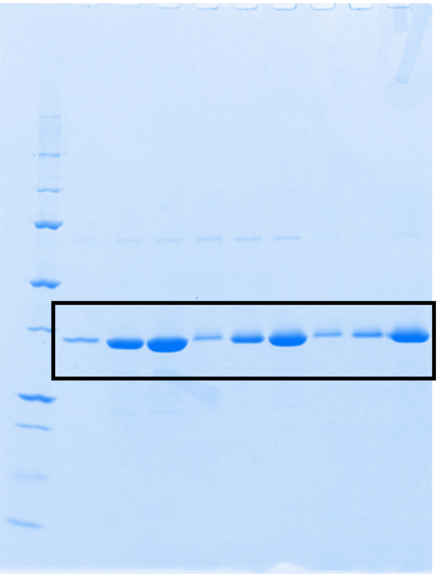

Figure 1c, row 2

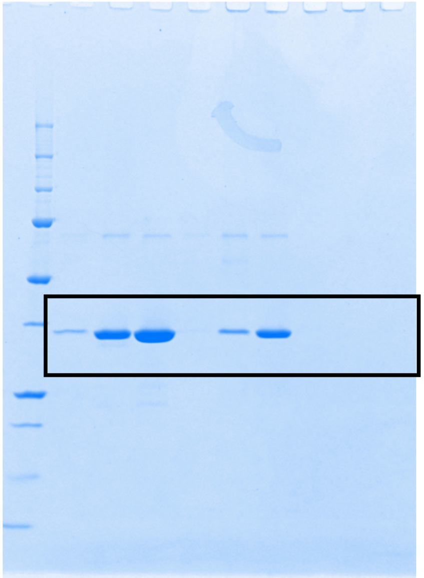

Figure 1c, row 3

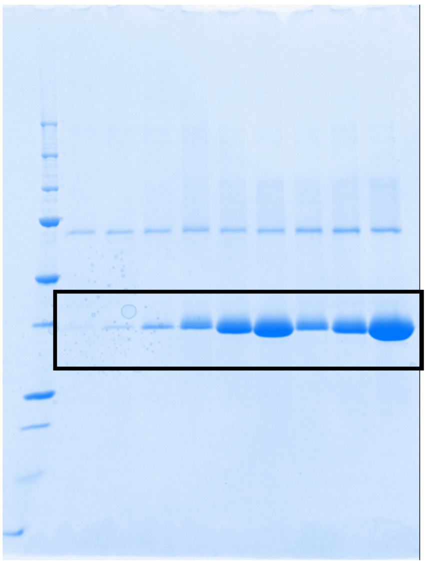

Figure 1b

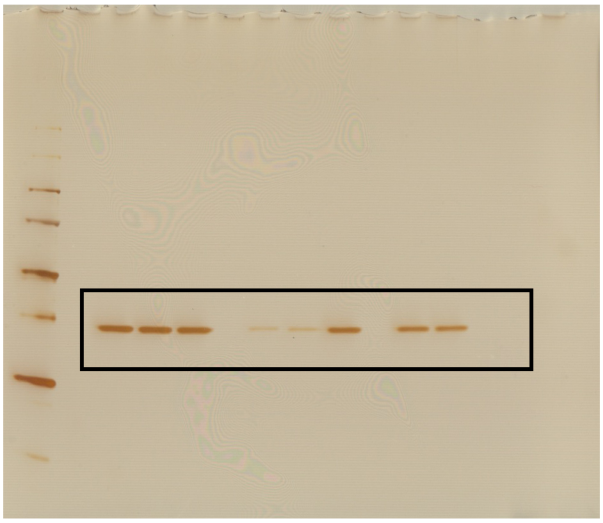

Figure 3b, row 1

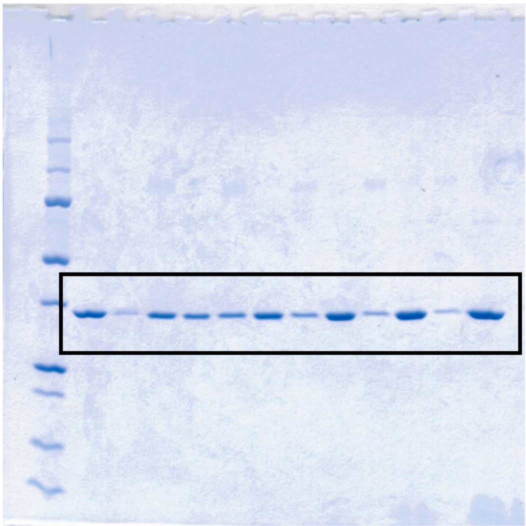

Figure 3b, row 3

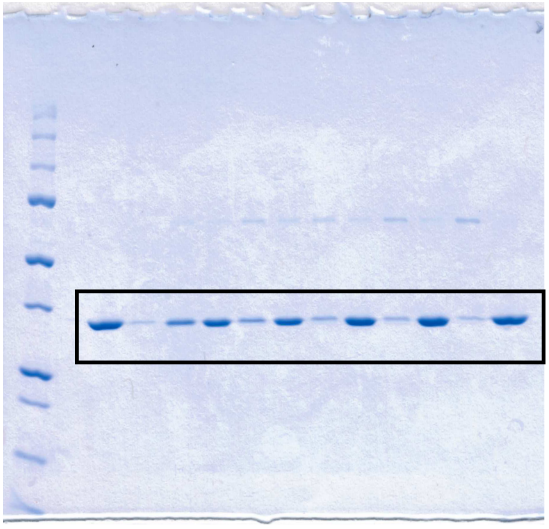

Figure 3b, row 2

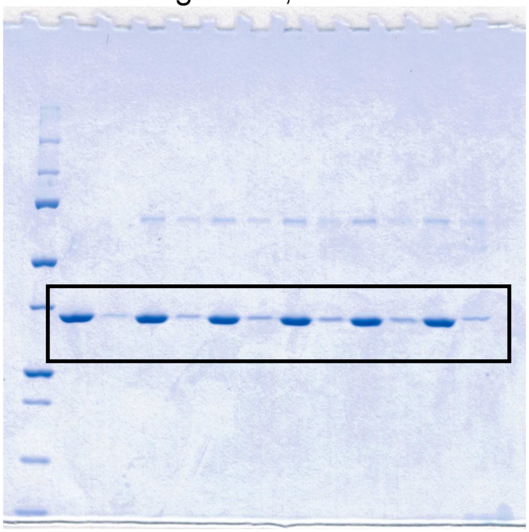

Supplementary Figure 13: Uncropped images of data shown in Figure 1b-c and 3b.

Supplementary Table 1. List of primers

	Forward primer (5'➔3')	Reverse primer (5'➔3')
**Primers for cloning**		
BoNT/A1	GGCCGGATCCATGCCATTTGTTAATAAACAATTTAATTATAA	GGCCGCGGCCGCTTACAGTGGCCTTTCTCCCC
tH_NA	GGCCGGATCCGCTTGAAGTCCTCTTTCAGGGACCCAAATATACTATGTTCCATTATCTTCGTGC	GGCCGCGGCCGCTTACTTAATATATTCAGTAAATGTAGATAATAATCTTTGATT
ciA-D12	GCCCGGATCCCAGGTGCAGCTCGTGGAGTC	GGGGGTCGACTCATTGTGGTTTTGGTGTCTTGGGTC
**Primers for mutagenesis**		
BoNT/A1^{F658E} or tH_NA^{F658E}	AGGTAATATGTTATATAAAGATGATTTTGTAGGTGCTTTAATAGAATCAGGAGCTGTTATTCTGTTAG	CTAACAGAATAACAGCTCCTGATTCTATTAAAGCACCTACAAAATCATCTTTATATAACATATTACCT
BoNT/A1^{S622C} or tH_NA^{S622C}	CCGATGAAACTAGCGAAGTATGTACTACGGATAAAATTGCG	CGCAATTTTATCCGTAGTACATACTTCGCTAGTTTCATCGG
BoNT/A1^{V653C} or tH_NA^{V653C}	GCTCCTGAAAATATTAAAGCACCACAAAAATCATCTTTATATAACATATTACCTATATTTAAAGCAGGTCC	GGACCTGCTTTAAATATAGGTAATATGTTATATAAAGATGATTTTTGTGGTGCTTTAATATTTTCAGGAGC
ciA-D12^{S124C}	GGTGTCTTGGGTCCACAGGAGACGGTGACCAG	CTGGTCACCGTCTCCTGTGGACCCAAGACACC

Supplementary Table 2. SEC-SAXS data collection and analysis

Data collection

Beamline	SSRL BL4-2
SEC system / column	Akta Ettan / Superdex-200 PC3.2/300
Sample volume	100 µl
Sample concentration	10.0 mg/ml
Sample buffer	55 mM HEPES (pH 7.0), 200 mM NaCl, 0.1 mM EDTA, 5 mM DTT
Flow rate	0.05 ml/min
Beam defining slits size	0.3 mm (H) x 0.3 mm (v)
Sample-Detector distance	1.7 m
Wavelength	1.127 Å (11 keV)
Beam current	500 mA (5 min top-off)
Exposure time	1 sec per 5 sec
Temperature	293 K
Sample cell size (quartz capillary)	1.5 mm in diameter

Guinier analysis

$q*R_g$ limit*	0.6-1.0
I(0)	2033.5 +/- 7.53
R_g	30.2 +/- 0.229 Å

P(r) and Porod volume estimation

Software	Primus/GNOM
q range*	0.02-0.35 $Å^{-1}$
I(0), real space	2052 +/- 3.80
R_g, real space	31.39 +/- 0.097 Å
D_{max}	117.5 Å
Porod volume	45300 $Å^3$
Calculated monomeric Mw (kDa) from sequence	35.6 kDa

ab initio modeling

Software (ab initio modeling)	DAMMIF
Software (averaging)	DAMAVER
q range*	0.18–0.35 $Å^{-1}$
Number of runs	20
Normalized Spatial Discrepancy (NSD)	0.602 +/- 0.011

*$q = 4\pi\sin(\theta)/\lambda$, where 2θ is the scattering angle.

Supplementary References

1. Bas, D. C., Rogers, D. M. & Jensen, J. H. Very fast prediction and rationalization of pKa values for protein-ligand complexes. *Proteins Struct. Funct. Genet.* **73,** 765–783 (2008).

2. Wimley, W. C. & White, S. H. Experimentally determined hydrophobicity scale for proteins at membrane interfaces. *Nature Structural Biology* **3,** 842–848 (1996).

3. Snider, C., Jayasinghe, S., Hristova, K. & White, S. H. MPEx: A tool for exploring membrane proteins. *Protein Sci.* **18,** 2624–2628 (2009).

4. Hofmann, K. & Stoffel, W. TMbase-a database of membrane spanning protein segments. *Biol. Chem. Hoppe Seyler* **347,** 166 (1993).

5. Cserzo, M., Wallin, E., Simon, I., von Heijne, G. & Elofsson, A. Prediction of transmembrane alpha-helices in prokaryotic membrane proteins: the dense alignment surface method. *Protein Eng. Des. Sel.* **10,** 673–676 (1997).

6. Persson, B. & Argos, P. Prediction of transmembrane segments in proteins utilising multiplesequence alignments. *J. Mol. Biol.* **237,** 182–192 (1994).

7. Lebeda, F. J. & Olson, M. A. Structural predictions of the channel-forming region of botulinum neurotoxin heavy chain. *Toxicon* **33,** 559–567 (1995).
